# Stress Monitoring Using Wearable Sensors: A Pilot Study and Stress-Predict Dataset

**DOI:** 10.3390/s22218135

**Published:** 2022-10-24

**Authors:** Talha Iqbal, Andrew J. Simpkin, Davood Roshan, Nicola Glynn, John Killilea, Jane Walsh, Gerard Molloy, Sandra Ganly, Hannah Ryman, Eileen Coen, Adnan Elahi, William Wijns, Atif Shahzad

**Affiliations:** 1 Smart Sensor Laboratory, Lambe Institute of Translational Research, College of Medicine, Nursing Health Sciences, University of Galway, H91 TK33 Galway, Ireland; 2 School of Mathematical and Statistical Sciences, University of Galway, H91 TK33 Galway, Ireland; 3 CÚRAM Center for Research in Medical Devices, University of Galway, H91 W2TY Galway, Ireland; 4 School of Psychology, University of Galway, H91 TK33 Galway, Ireland; 5 Electrical and Electronic Engineering, University of Galway, H91 TK33 Galway, Ireland; 6 Centre for Systems Modelling and Quantitative Biomedicine (SMQB), University of Birmingham, Birmingham B15 2TT, UK

**Keywords:** stress-predict dataset, photoplethysmogram (PPG), biomedical signal processing, adaptive reference ranges, non-invasive devices, health monitoring, heart rate, respiratory rate

## Abstract

With the recent advancements in the field of wearable technologies, the opportunity to monitor stress continuously using different physiological variables has gained significant interest. The early detection of stress can help improve healthcare and minimizes the negative impact of long-term stress. This paper reports outcomes of a pilot study and associated stress-monitoring dataset, named the “Stress-Predict Dataset”, created by collecting physiological signals from healthy subjects using wrist-worn watches with a photoplethysmogram (PPG) sensor. While wearing these watches, 35 healthy volunteers underwent a series of tasks (i.e., Stroop color test, Trier Social Stress Test and Hyperventilation Provocation Test), along with a rest period in-between each task. They also answered questionnaires designed to induce stress levels compatible with daily life. The changes in the blood volume pulse (BVP) and heart rate were recorded by the watch and were labelled as occurring during stress-inducing tasks or a rest period (no stress). Additionally, respiratory rate was estimated using the BVP signal. Statistical models and personalised adaptive reference ranges were used to determine the utility of the proposed stressors and the extracted variables (heart rate and respiratory rate). The analysis showed that the interview session was the most significant stress stimulus, causing a significant variation in heart rate of 27 (77%) participants and respiratory rate of 28 (80%) participants out of 35. The outcomes of this study contribute to the understanding the role of stressors and their association with physiological response and provide a dataset to help develop new wearable solutions for more reliable, valid, and sensitive physio-logical stress monitoring.

## 1. Introduction

Stress is known as a silent killer that contributes to several life-threatening health conditions such as high blood pressure, heart disease, and diabetes. According to the British Health and Safety Executive, 50% of all work-related illnesses in 2021–2022 were due to stress [1]. Stress has negative effects on the mental health as well as the overall well-being of a person [2]. Short-term stress may not impose any threat to young and healthy people who have an adaptive coping response, but if the stressful experience is too persistent or too strong, it may increase the risk of developing chronic conditions associated with depression and anxiety [3]. Long-term stress is also known to increase the risk of life-threatening illnesses such as heart disease, high blood pressure, diabetes, and obesity while an acute episode of stress can potentially trigger a heart attack or stroke [4]. In clinical settings, the subjective experience of stress is evaluated from psychometric methods such as self-reported questionnaires, e.g., the Perceived Stress Scale (PSS) [5] and/or State-Trait Anxiety Inventory (STAI) [6].

To develop a reliable objective stress monitoring device, it is essential to understand the effects of stress from the perspective of changes in the relevant physiological and biochemical variables. During standardized stress-inducing procedures, the sympathetic nervous system of the body is triggered, causing the release of different hormones (like cortisol or adrenaline) [7,8]. These hormones lead to changes in respiratory rate and heart rate, and trigger muscle tension among other physiological responses that prepare the body for fight or flight reactions. Both physical and biochemical changes can be used as indicators of stress and measured using different wearables. Some real-time stress-monitoring devices/models are described in Refs. [9,10,11,12,13,14]. There are multiple reasons behind the lack of a reliable objective stress-monitoring device/model., the foremost of which is the absence of a universally acceptable definition of stress. Moreover, the lack of gold standard ground-truth/reference values or data, collection of stress data in the natural environment, different confounding variables, identification of discriminative/specific stress features, and development of an accurate classifier model to classify stress data from baseline/normal are also contributing reasons to the lack of unswerving stress monitoring device. Further details are explained in [15].

### 1.1. Related Work

The proposed study is inspired by several existing works in the field of wearable devices for stress detection and monitoring. The WESAD (Wearable Stress and Affect Detection) dataset [16] was created using RespiBAN and Empatica E4 as wearables. The authors monitored the stress levels of 15 students while they were watching movies and taking a trier social stress test (TSST). The random forest classifier achieved an accuracy of 75.2% using blood volume pulse (BVP), electrodermal activity (EDA) and temperature readings while distinguishing between three classes (baseline vs. stress vs. amusement). The SWELL-KW dataset [17] used video (for facial expression), computer logging, and Kinect (3D sensor for body posture) to monitor the stress levels of 25 people while they were performing typical knowledge work (making a presentation, reading, writing reports, email) under time pressure. The author reported averaged subjective experience scores using task load, mental effort, emotion, and perceived stress questionnaires for all the subjects. The study concluded that, based on subjective scores, there was no significant effect of work conditions on perceived stress levels. The Affective-Road dataset [18] used Zephyr Bioharness 3.0 and Empatica E4 to study the stress levels under different driving conditions for 10 drives. The data were collected during driving for 1 h 26 min on different types of roads and under different traffic conditions, and no statistical or classification analysis was performed on the dataset. The author suggested that their prototype provides an accurate collection of different signals. Thus, vehicle manufacturing companies can embed the system into their vehicle and provide a real-world experimental dataset for studying the effect of road type on drivers’ stress levels. Healey et al. [19] developed a wearable glove with an embedded photoplethysmogram (PPG) sensor to monitor the stress levels of 10 drivers while they drove on different routes. Stress vs. normal-state classification was performed using electrocardiogram (EKG), electromyography (EMG), Respiratory rate and galvanic skin response (GSR) signals. The accuracy of 62.2% was reported by the authors using a sequential forward floating selection (SFFS) k-NN classifier. Shi et al. [20] developed a multi-node stress monitoring system based on ECG, EDA and PPG signals. They collected data from 22 subjects and reported that a support vector machine (SVM) model gave the highest accuracy of 68% when distinguishing stressed conditions from normal. Similarly, Muaremi et al. [21] were able to detect different stress levels using a smartphone and Wahoo wearable chest belt. They experimented on 35 subjects, collecting heart-rate variability and smart phone application (questionnaires) data for 4 months. The combination of this information resulted in the highest three stress level (low, moderate and high) classification accuracy of 61% using logistic regression-based leave-one-outcross-validation. Hosseini et al. [22] created a multi-sensor dataset of nurses working in the hospital during the COVID-19 outbreak. They used Empatica E4 watches to collect information about the electrodermal activities, heart rate, and skin temperature of the subjects. The authors concluded that the device was unable to detect physiological differences across the various stress exposures.

From the literature review, it can be concluded that the optimal measurement approach for physiological stress-monitoring is still unclear. Different studies have used the same physiological variables and classifiers but have reported significantly different classification accuracies. Furthermore, there is no clear understanding of the relative sensitivity and specificity of stress-related biophysiological indicators of stress (such as heart rate and respiratory rate) in the literature [15,23]. All the above-mentioned datasets have a relatively small sample size and are more focused on performing classification analysis with reported accuracies in the range of from 60 to 70% rather than performing a statistical analysis of the dataset. These analyses are critical in understanding the relative importance of the most common and clinically relevant physiological stress indicators, as well as in identifying the most specific indicators of stress for the development of a reliable stress-monitoring device.

### 1.2. Study Objectives

The accurate monitoring of physiological stress levels has the potential to assist physicians in guiding their patients to adapt their lifestyle decisions, e.g., individual occupational contexts, inform personalised treatment plans, and ultimately improve their overall health. Therefore, this study aims to develop a *stress-predict dataset* and perform statistical analysis of biophysiological data collected from healthy individuals who underwent induced psychological stress to assess the relative sensitivity and specificity of common biophysiological indicators of stress and provide a stepping-stone towards the development of an accurate stress monitoring device. In this study, 35 healthy volunteers performed three different stress-inducing tasks (i.e., Stroop colour word test, Trier Social Stress Test and Hyperventilation Provocation Test session) with a baseline/relax period in-between each task. Blood volume pulse (BVP), inter-beat-intervals (in milliseconds), and heart rate (in beats per min) were continuously recorded using Empatica watches. The key objectives of the study are as follows:

- Collect physiological data for wearable stress monitoring (stress-predict dataset).
- Perform statistical analysis and analyse the dataset to study association of various physiological variables and stress levels.
- Assess the effectiveness of stress-inducing activities for experimental studies.

### 1.3. Key Contributions

The key contributions of this study are as follows:

- Collected PPG signals using an Empatica E4 watch (a wrist-worn device) and developed an open-access dataset.
- Estimated respiratory rate readings from the raw signal using a novel PPG-based respiratory rate estimation algorithm [24] and included them in the dataset.
- Performed individual-level statistical analysis using a novel method based on the Bayesian framework and time-efficient approximate Expectation-Maximisation (EM) algorithm [25].

The rest of the paper is organised as follows: Section 2 provides an overview of the proposed protocol and data analysis metrics; Section 3 presents details of data features included in the dataset; a detailed analysis and results are provided in Section 4; Section 5 concludes the paper, discusses protocol limitations and provides future directions towards the development of an accurate stress-monitoring device.

## 2. Material and Methods

### 2.1. Study Design

This was a research study aimed at providing useful information and facts on stress in healthy individuals from data recorded using a wrist-worn watch. The study was a quasi-experimental repeated measures design where participants were assessed across a set of standardised psychological stress induction protocols over a 60-min laboratory-based testing session. There was no longer-term follow-up on the participants. This was an opportunity to sample from a healthy individual population.

### 2.2. Selection and Recruitment of Participants

All study participants were selected and consecutively enrolled in the study based on inclusion/exclusion criteria specified in Table 1. If the participant was eligible for inclusion and informed consent is obtained, the participant was entered onto the study enrolment log and assigned a unique subject ID number. This healthy volunteer study was advertised via brochures and posters at University Hospital Galway (UHG) and the University of Galway. The clinical research team also helped to recruit volunteers. The study protocol and patient information consent forms were approved by the local Ethics Committee (on 19 January 2022 *Ref: C.A. 2731*).

### 2.3. Study Methodology and Protocol

The study, adapted from [26], was completely non-invasive and took approximately 60 min to complete for each participant. The consent form was given to the interested participant, who was given sufficient time (2 days) to read, understand, and ask any question to the Lead Researcher/Investigator. During this period, the participants had to decide whether they wanted to participate in a research study. All participants were asked to read and sign the consent form before the start of the study. Moreover, at the beginning of the experiment, each participant was reminded of the order of phases, the duration of each phase, and what they were required to do in each phase.

**Figure 1:**
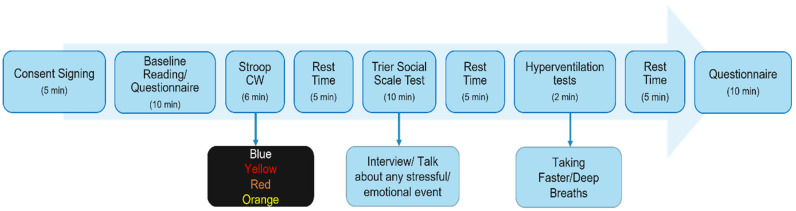
Study Protocol of the stress-monitoring study including 3 stress-inducing tasks/sessions, 2 self-reporting questionnaire sessions and in-between rest sessions.

It is well established that social evaluative acute stressors such as the colour word (Stroop-CW) test and the Trier Social Stress Test elicit the strongest physiological responses in laboratory settings when compared to cognitive challenges [27,28]. There is also the argument that conducting interviews is a more ecologically valid analogue of the real-world social stressors in which we are interested [29]. The two questionnaires (PSS and STAI) are the most popular ways of assessing stress [9,30,31,32]. These questionnaires help us to understand how different situations affect participants’ feelings and anxiety. Furthermore, to estimate the respiratory rate from the PPG signal, a 2-min Hyperventilation Provocation Test task was also performed to obtain a reference reading. After each task, the participant was asked to relax for 5 min. The following protocol, illustrated in Figure 1, was followed in the proposed study.

Stress-inducing tasks might induce some degree of lasting stress. If participants felt stressed during or after the study, the research team, including clinical nurses, made sure that they had enough time to relax before starting a new task or going home. Furthermore, the participants were instructed to contact Clinical Research Facility Galway, University Hospital Galway or Student Health Unit, the National University of Ireland Galway in the event of persistent stress.

### 2.4. Study Sample Size Calculation

Previously, in a detailed literature survey and statistical analysis to determine the most sensitive and specific parameters for stress-monitoring, we concluded that the respiratory rate (RR) is the most important parameter for the detection of stress conditions [15,16,33]. The results of these statistical analyses were published in [15]. For this study, an easier and quicker option would be to have power for a paired sample comparison, i.e., comparing RR within individuals when they are stressed vs. not stressed. We have a within-person or paired design, as each person will undergo periods of stress and no stress.

A clinically significant difference in stressed vs. unstressed respiratory rate is a 10% difference [34]. The control respiratory rate and variability reported in [15] was 12.35, so a 10% increase is 13.58. The variability in Respiratory Rate from the same paper was 2.5. Using these summary statistics, a sample size of *n* = 34 participants is required to achieve 80% power to detect a 10% change in RR, at the alpha 0.05 significance level. Thus, a total of 35 healthy volunteers (females and males) aged between 18 and 75 years old were recruited for the study to allow for attrition.

### 2.5. Data Acquisition

**Figure 2:**
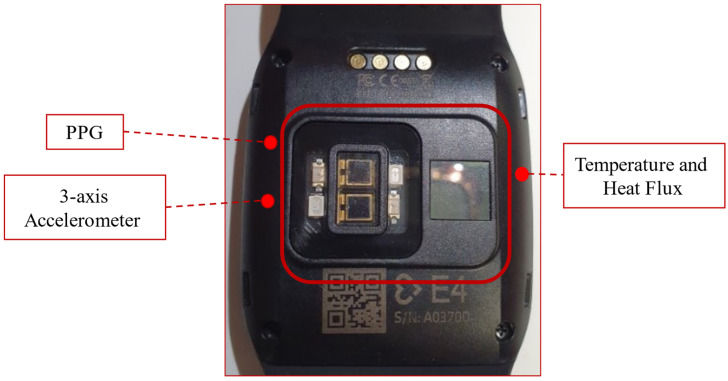
Empatica E4 watch.

In this study, an Empatica E4 watch (Figure 2, adopted from [35]) was used to measure individual physiological changes based on PPG, which was previously used in several similar studies [16,18,22,36,37,38,39]. The watch is a medical-grade device that is classified as Class IIA Medical Device according to the 93/42/EEC Directive. Empatica E4 is a wireless multi-sensory platform designed to acquire real-time physiological data with ease.

#### Empatica E4 Photoplethysmogram (PPG) Sensor

The PPG sensor embedded in the watch has a sampling rate of 64 Hz. The raw PPG signal is filtered to obtain a clean blood volume pulse (BVP) signal, which is then passed to the heart rate (HR) and inter-beat intervals (IBI) estimation algorithm. There are 2 green and 2 red light-emitting diodes (LEDs) that transmit light onto the skin. To receive the reflected light, there are 2 photodiodes in-place with an area of sensitivity of 14 mm^2^.

The output of the PPG sensor is digital and has a resolution of 0.9 nW/Digit. Exposure to green light contains information about heartbeats, whereas exposure to red light assists in reducing noise or motion artefacts by dynamical compensation performed by the built-in firmware. The accuracy of Empatica E4 heart rate readings are highly comparable with standard ambulatory monitory system. A detailed comparison of Empatica E4 readings with ambulatory monitoring systems is provided in [40].

The participants were asked to wear the watch on the non-dominant wrist. The watch can be operated in memory mode. In this mode, the data are stored on the built-in memory of the watch and once the session is completed, the data are uploaded to the ’Connect’ cloud via personal computer or laptop using Empatica E4 manager software. Data can be visualized in the cloud for visual analysis and could be downloaded from the cloud in .csv format. The data of each sensor, as well as estimated heart rate and inter-beat-intervals, are downloaded as separate files. In our case, the start and end of each task were labelled by clicking once the button on the watch, thus, along with the physiological data, corresponding tags were also generated.

To induce stress, the participant performed the Stroop Colour-Word task, Trier Social Stress Test, and Hyperventilation Provocation Test tasks. From the start of the experiment to the end of the recording, each section was labelled using a built-in function (pressing the button on the watch once). Labelling helped us identify whether any stressor had a prolonged reaction even after the stimulus.

### 2.6. Data Analysis Matrices

Two statistical analyses are used to determine the utility of the stress-predict dataset:

(i) Linear Mixed Model analysis

A linear mixed model was implemented for population-based analysis to determine the effect of stressors on HR and RR, while accounting for the correlation of these variables within each person over time. A separate model was run for RR and HR, with random intercept and slopes included for each participant to allow for within- and between-subject variability. The binary group variable is included as a fixed effect, and the coefficient of this variable in the model describes the average difference in the result between stress and normal situations. An interaction term between time and group (stress/normal) is also included, with the coefficient of this providing an estimate for the difference in change in outcome over time between stress and normal situations. Results are reported as coefficients, with 95% confidence intervals and *p*-values from linear mixed models.

(ii) Adaptive reference range analysis

For the development of an extensive understanding of changes in participants’ response over the study time, an individual-level statistical analysis was performed by the development of personalised adaptive referencing ranges, proposed by Davood et al. [25]. In this method, to see if there are any meaningful changes in a particular participant’s response over time, individualised reference ranges are developed, which successively adapt whenever a new measurement is recorded for the individual. In the context of this work, adaptive reference ranges were generated sequentially according to normal physiological rates at each resting phase, and then the following stress data were included for comparison. Any value outside the developed adaptive reference range can be considered an ‘alert’ that requires further consideration. The adaptive referencing range method works using a Bayesian framework and time-efficient approximate Expectation-Maximisation (EM).

## 3. Data Features Included in Stress-Predict Dataset

The created dataset consists of physiological data collected from 35 students and employees of the University of Galway, Ireland, and the University Hospital Galway, Ireland. The participant performed three stress-inducing tasks, along with four rest periods. All the readings have been tagged as the duration of the stress-inducing task and baseline/rest period. The time of each tag is available in the tag.csv file in the dataset. In all other files, the first row shows the timestamps in Unix timestamp UTC format, while the second column shows the sampling rate in Hz. The collected data start from the third row and continue until the end. The data in the given Stress-Predict dataset are expressed as:

### 3.1. Blood Volume Pulse

**Figure 3:**
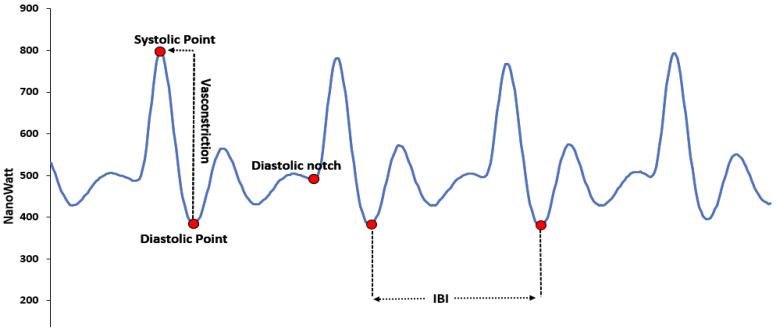
PPG signal obtained in typical condition, from the green and red light.

This file has data collected by a PPG sensor. Typically, a BVP signal is obtained by passing the PPG through a high-pass filter. The cut-off frequency of this filter can be arbitrary but typically set between 0.05 and 0.5 Hz. The data in the file represent the BVP value calculated by the built-in algorithm in units of nanowatt units (nWatt). Figure 3, adapted from [41], shows the typical PPG signal and its significant points.

In the PPG signal:

- The diastolic point is the local minima point, used to calculate the inter-beat-interval.
- The systolic point is a local maxima point, used to calculate the vasoconstriction of the participant.
- The presence of a dicrotic notch is observed in the study of different types of cardiac diseases.
- The dicrotic wave is the effect of the dicrotic notch and is referred to as the second wave.

### 3.2. Inter-Beat-Intervals

**Figure 4:**
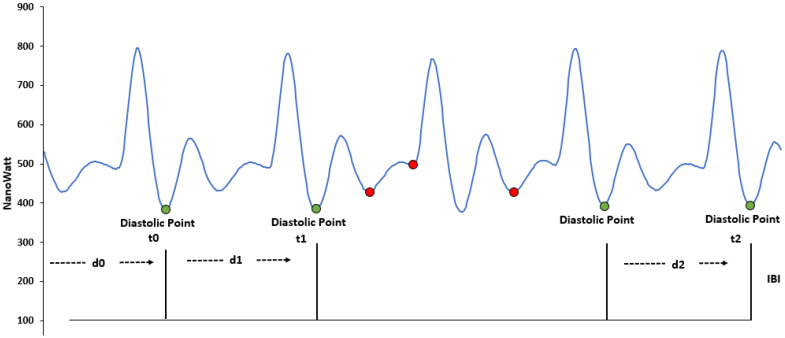
Inter-beat-intervals calculation. The green dots show valid peaks while red dots show the discarded peaks.

The file contains time intervals between two consecutive heartbeats. The IBI values in the dataset are obtained by processing the BVP signal, with an algorithm that already eliminates the incorrect peaks in the signal generated due to noise. Figure 4 shows the PPG/BVP signal with some motion artefacts. The green dots show correct heartbeats while the red dots show incorrect heartbeats, corresponding to the time of movement. The timing of incorrect beats is not included in the IBI file, as demonstrated in Figure 4 (adapted from [42]). The first row shows the timestamps in UNIX format, while the first column (excluding the first row) illustrates the time of detected inter-beat-intervals in seconds (s). The second column shows the distance in seconds (s) from the previous beat (the detected IBI); see Table 2.

### 3.3. Heart Rate

The file has average heart rate values calculated by the watch from the raw BVP signal. The heart rate in this file is calculated with a 10-s sliding window. This is only created when the session is completed and uploaded to the Empatica *‘connect’* cloud. The unit of each heart rate is beats per minute (BPM). Instantaneous heart rate can only be viewed during stream mode or online (in view session).

### 3.4. Labels

The file contains time marks when an event is marked. Each row corresponds to the physical button pressed on the watch. The time is presented in form of Unix timestamps in UTC and is synchronized with the initial time of the session indicated in the related data file.

### 3.5. Estimation of Respiratory Rate Data

The created dataset is different from all existing public datasets, as it included information on the estimated RR for each participant. As explained in [24], the study proposed a novel RR-estimating algorithm that worked on raw PPG signals. As BVP is a filtered form of raw PPG signal, the developed algorithm was able to estimate the RR of the participants during stress, as well as rest/baseline time. The algorithm was implemented in three-fold steps (pre-processing, signal analysis, and post-processing).

**Figure 5:**
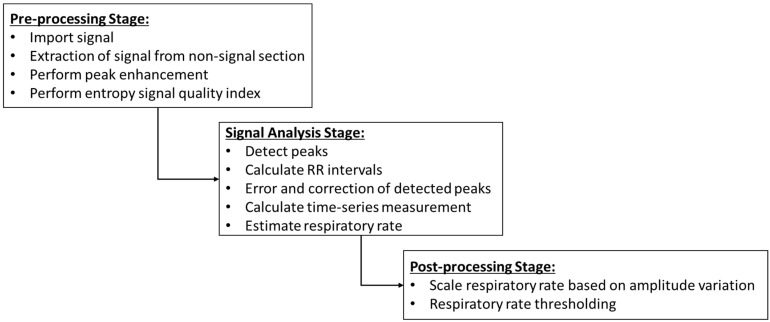
Pre-processing, signal analysis and post-processing steps of the RR estimation algorithm.

Figure 5 explains the steps of the proposed RR estimation algorithm. In the pre-processing stage, peak enhancement was performed to increase the signal-to-noise ratio and ensure better signal information extraction. Peak detection, peak-to-peak interval, error and correction in peak detection, calculation of time-series measurement and estimation of RR were carried out during the signal analysis stage. Usually, the BVP waveform is synchronised with the respiratory cycle [24]; thus, an amplitude variation is induced in the raw signal. In the post-processing stage, the estimated RR is scaled based on the range (maximum-minimum value) and defined window size of the signal. The data of the estimated respiratory rate are included in the dataset as a separate file.

## 4. Analysis and Results

There were 25 women and 10 men participants (mean age = 32 ± 8.2 years) in the created dataset. The data collection protocol was not followed properly for one (1) participant, and their data were removed from further analysis. The average number of entries per participant is presented in Table 3.

**Figure 6:**
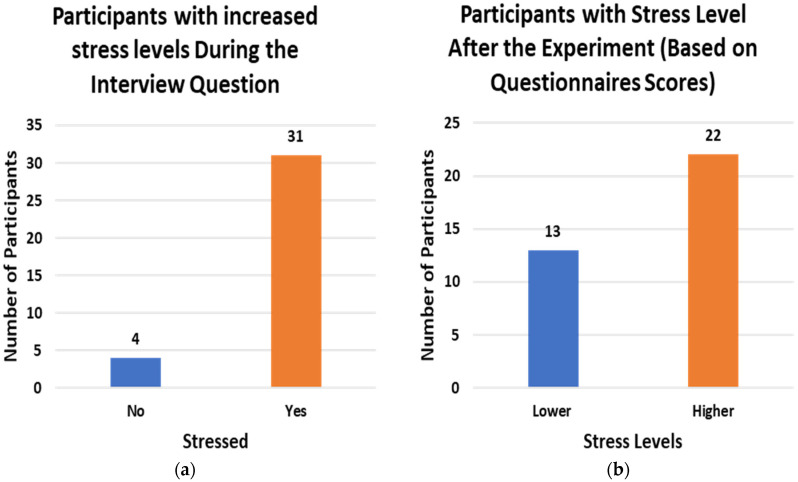
Participants with increased stress levels (**a**) based on Questionnaire score (**b**) asked during Interview.

During the study, the participants were asked to fill out STAI and PSS questionnaires to rate their stress levels and were also asked during the Trier Social Stress Test whether they felt stressed at any point of the study. Figure 6 shows the number of participants with increased stress levels after the study (a) based on questionnaire scores and (b) based on the verbal query.

### 4.1. Population-Based Analysis Using Linear Mixed Model

According to the results in Table 4, during stress state, the HR was 1.40 beats per minute higher on average compared to normal state HR (95% CI 1.10, 1.71; *p* < 0.001). Participants also experienced a 5.05 bpm higher change in HR per hour compared to during a normal state (95% CI 4.36, 5.74 bpm/h; *p* < 0.001).

When exposed to stress, participants’ average RR increased by 0.20 breaths per minute compared to their normal state (95% CI 0.16, 0.24 breaths per minute; *p* < 0.001), see Table 5. Participants also experienced a −1.11 breaths per minute lower change in respiratory rate per hour compared to during a normal state. The drop in the RR can be related to sighs (deep breaths when under stress).

Although the change in average HR and RR during the stress period is statistically significant (*** *p* < 0.001) when compared to the average value of the normal/baseline state, the difference may not be large enough for clinical decision-making.

### 4.2. Individual Participant’s Analysis Using Adaptive Reference Range

In this method, each stress period is compared with the last reference range generated from the previous normal period to allow for the early detection of abrupt physiological changes.

**Figure 7:**
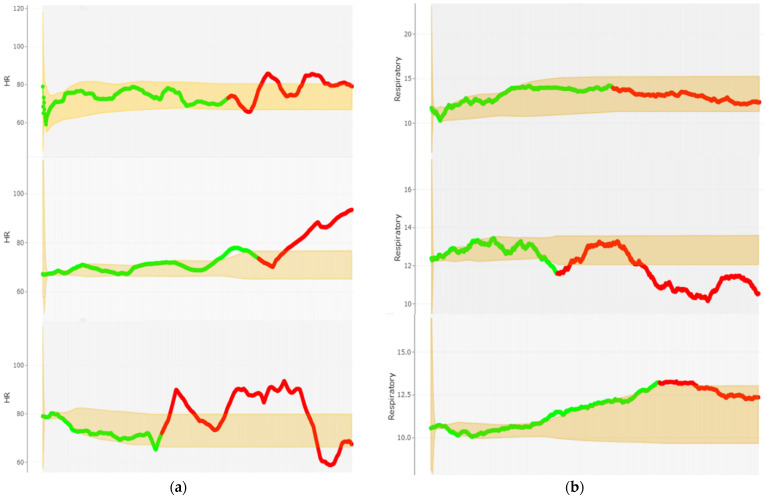
Statistical Analysis of Participant 23: Adaptive referencing range (shaded region) calculated by using approximate EM. (**a**) Heart rate reading: baseline (green) vs. stress (red) task (**b**) Respiratory rate: During each baseline (green) vs. stress (red) task.

Figure 7a,b illustrate the developed reference ranges for the HR and RR of participant 23, respectively. As can be seen from Figure 7a, there are a number of atypical HR measurements for participant 23 in all three phases of this study. This is particularly true for the two Trier Social Stress Test and Hyperventilation Provocation Test sessions. This is where none of the respiratory rates go outside the developed reference range during the Stroop color test session, Figure 7b. For figures of all other subjects, see *Sfig1_statistical_analysis_HR_plots* and *Sfig2_statistical_analysis_RespR_plots* in the Supplementary Material.

It should be noted that the developed adaptive reference ranges are not a classification algorithm but are capable of triggering ‘alerts’ and should be used as an early warning system that warrant further attention and review.

Summative results of individual participant’s analysis on HR and RR parameters presented in Table 6.

## 5. Discussion and Conclusions

The Stress-Predict dataset was developed using a commercially available wearable E4 watch by Empatica [43]. The purpose of developing this dataset was to analyse and identify different patterns of stress.

Most stress detection and monitoring studies report only the classification results and lack a statistical analysis of the extracted features. Moreover, the studies that report statistical analysis results perform a group analysis (considering all participants as a single group). The limitation of the group analysis is that, within a group (each participant), the variability of stress-related parameters is quite high. For example, the normal heart-rate values of one participant could overlap with the stressed heart-rate value of another participant, and vice versa. The group analysis exploits this variability, and thus results in biased outcomes. In this study, an individual analysis was also performed, along with a group analysis (linear mixed model), to obtain ore insightful information. To validate that this difference in the readings is significant, the linear mixed model was implemented for group analysis while the development of personalized adaptive reference range allowed for individual-level monitoring of heart rates and respiratory rates. Both the models validated the hypothesis that the physiological data collected during stress and non-stress/baseline task are statistically differentiable. Table 7 provides a comparison of proposed dataset with the state-of-the-art publicly available dataset.

The dataset is an open-access dataset named Stress-Predict dataset. The inclusion of an additional feature, i.e., respiratory rate data, along with stress and baseline labels within the dataset, makes the dataset more desirable and unique from all the other publicly available Empatica E4-based datasets. Additionally, the developed dataset will also help to evaluate proposed PPG-based feature extraction algorithms. The current dataset will certainly attract the attention and interest of researchers in the field of psychological, clinical, and biomedical research, as well as prevention, medicine, and connected health systems.

There were also some limitations to the study. First, at the start and end of the stress task, the HR and RR gradually changed, but there is no accurate way to determine this gradual change. Thus, labelling was performed without considering these delayed changes. Secondly, sometimes there was more than one participant in the room where the study was conducted. The crosstalk, and especially the questions asked during the Trier Social Stress Test to induce stress, might be learned by the other participant during the resting/baseline period. Therefore, the effectiveness of the stress-inducing interview questions could have been decreased. Third, the interviewees were friendly and kind to the participant. They kept the overall interview environment friendly rather than mimicking a strict interview session, which might have resulted in less induced stress. Thus, there was less variation in the readings of stress versus non-stress parameters. To translate the proposed model to an ambulatory environment, the inclusion of activity data is also essential. In future studies, all these shortcomings should be considered to obtain an improved stress-monitoring model. Moreover, to obtain an accurate real-time stress monitoring system, accelerometer data might play an essential role in excluding the period of physical exercise, which causes changes in HR and RR. In future, the developed dataset might help in exploring, optimizing, and developing supervised and unsupervised machine-learning classifiers for the detection of physiological signal-based stress monitoring.

## Figures and Tables

**Table 1:** Selection criteria.

Inclusion Criteria	Exclusion Criteria
Healthy (no underlying health condition)	No consent
Age between 18 and 75 years	Unhealthy
English speaking (all ethnicities)	Breastfeeding mothers, pregnant women
Give consent	Colour-blind

**Table 2:** Inter-beat-intervals in IBI.csv file (unit = µS).

UNIX Start t	IBI
t0	d0
t1	d1
t2	d2

**Table 3:** The average number of entries (per participant).

Features	Samples
Blood Volume Pulse (BVP)	212,234
Heart Rate (beats per min)	3308
Respiratory Rate (breaths per min) [Calculated using sliding window of 10 s]	3308

**Table 4:** Linear Mixed Model Results for Heart Rate Parameter.

Predictors	Estimates	Confidence Intervals		*p*-Value
		Lower	Higher	
(Intercept)	80.36	76.85	83.88	<0.001
Time	−2.65	−5.85	0.55	0.105
Group [Stress]	1.40	1.10	1.71	<0.001
Time * Group [Stress]	5.05	4.36	5.74	<0.001
Observations	112,472			

**Table 5:** Linear Mixed-Model Results for Respiratory Rate Parameter.

Predictors	Estimates	Confidence Intervals		*p*-Value
		Lower	Higher	
(Intercept)	12.68	11.98	13.39	<0.001
Time	−0.30	−1.02	0.41	0.408
Group [Stress]	0.20	0.16	0.24	<0.001
Time * Group [Stress]	−1.11	−1.19	−1.03	<0.001
Observations	112,472			

**Table 6:** Summary of Statistical Analysis (adaptive reference range).

Heart Rate	Respiratory Rate	Heart Rate
Test	Stress (Outside Baseline Values)	Stress (Outside Baseline Values)
Stroop Test	24/34	19/34
Trier Social Stress Test	28/34	27/34
Hyperventilation Provocation Test	18/34	16/34

**Table 7:** Summary: Comparison of Proposed Dataset with Existing State-Of-The-Art Datasets.

Study	Devices Used	No. of Subjects	Methods	Features	Limitations	Pros
[16]	RespiBAN and Empatica E4	15	BVP, EDA, EMG and Temperature sensors	Heart rate, skin conductance, respiratory rate, muscle activation and skin temperature	Uses chest band Subjects must remain immobile Not a translational (practical) model (use of chest band) No justification of selected sample size	Data gather through chest band is highly accurate Respiratory rate data obtained by chest band
[17]	Video camera, computer logging and Kinect device	25	Task load, mental effort, emotion, and perceived stress questionnaires	Facial expression, computer logging and 3D body posture monitoring	Need control environment Not a translational (practical) model (3D Kinect) No justification of selected sample size	Provided subjective (personalized) results (stress versus work condition)
[18]	Zephyr bio harness and Empatica E4	10	EDA, temperature, BVP, camera	Skin conductance and temperature, heart rate, respiratory rate, and hand movements	Not a translational (practical) model (use of chest band) No justification of selected sample size	Data gathered through chest band are highly accurate Respiratory rate data obtained by chest band
[21]	Smart-phone and Wahoo chest belt	35	Number of calls, sleep length, distance, audio length, heart rate variation	Inter-beat-inter/heart rate	Not a translational (practical) model (use of chest belt) No justification of selected sample size	Big data (4 month)
This Work	Empatica E4	35	BVP (wrist band)	Heart rate and respiratory rate	Limited (approx. 60 min) data Small subject age window	A translational (practical) model Justification of selected sample size Only Empatica E4 dataset with respiratory rate data Provides subjective outcomes

## Data Availability

Raw data supporting the conclusions of this manuscript is made available by the corresponding author at https://github.com/italha-d/Stress-Predict-Dataset (accessed on 3 October 2022).

## Supplementary Materials

The following supporting information can be downloaded at: https://www.mdpi.com/article/10.3390/s22218135/s1, HR_Plots and RespR_Plots.
