# Supplementary material for: Stress Monitoring Using Wearable Sensors: A Pilot Study and Stress-Predict Dataset
Source: Sensors (Basel). 2022 Oct 24;22(21):8135. doi: 10.3390/s22218135 (PMC9654418; doi:10.3390/s22218135)
Supplement: Supplementary file 1 [file sensors-22-08135-s001.zip › RespR_Plot.pdf]

P2:

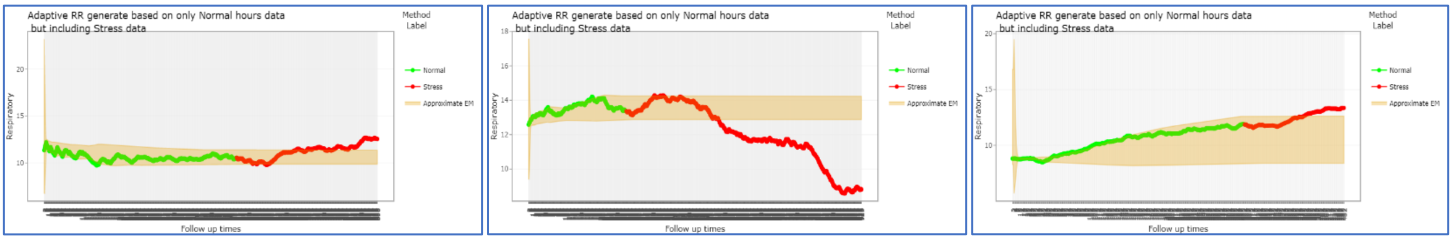
Adaptive RR generate based on only Normal hours data
but including Stress data
Method
Label
Normal
Stress
Approximate EM
Respiratory
Follow up times

P3:

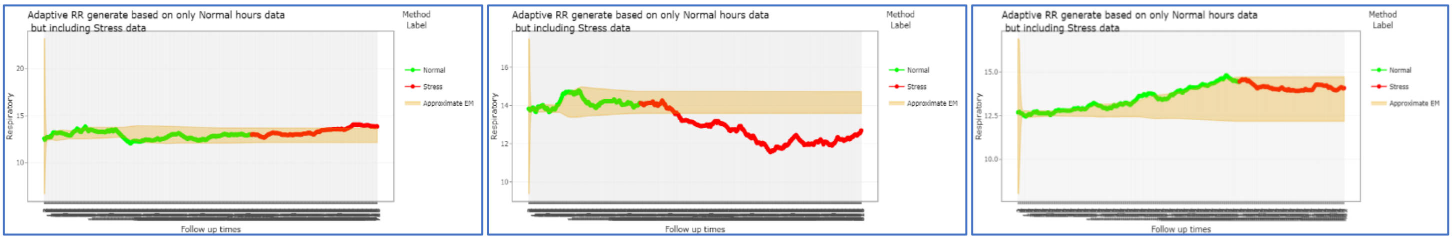
Adaptive RR generate based on only Normal hours data
but including Stress data
Method
Label
Normal
Stress
Approximate EM
Respiratory
Follow up times

P4:

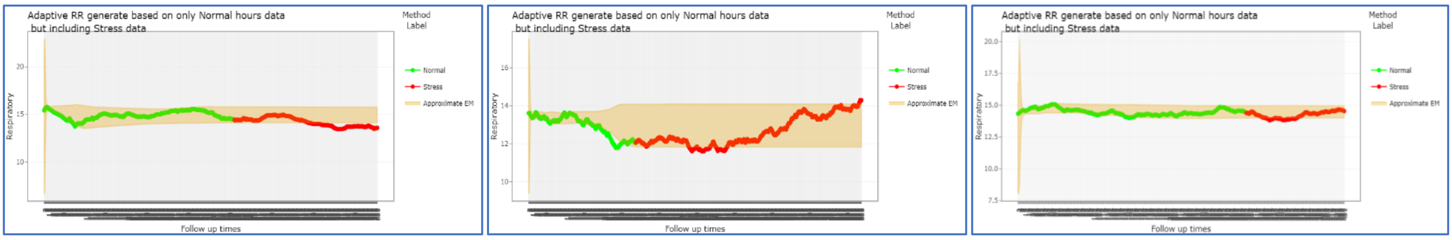
Adaptive RR generate based on only Normal hours data
but including Stress data
Method
Label
Normal
Stress
Approximate EM
Respiratory
Follow up times

P5:

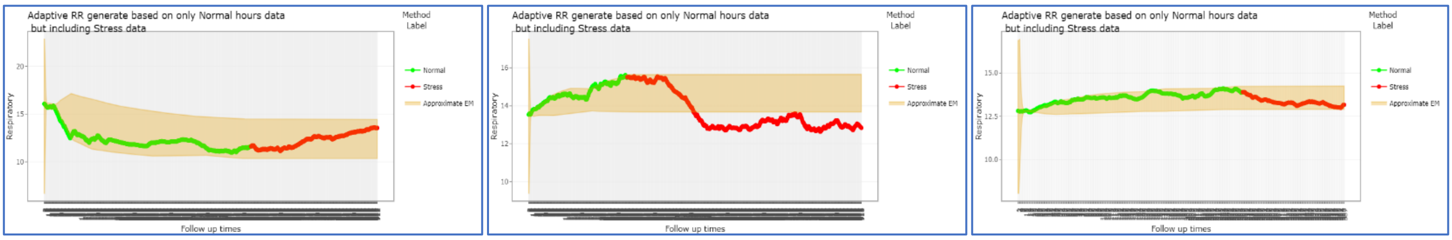
Adaptive RR generate based on only Normal hours data
but including Stress data
Method
Label
Normal
Stress
Approximate EM
Respiratory
Follow up times

P6:

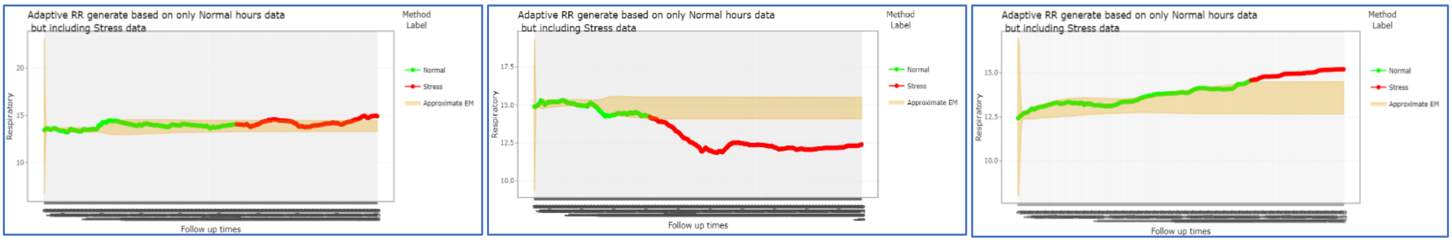
Adaptive RR generate based on only Normal hours data
but including Stress data
Method
Label
Normal
Stress
Approximate EM
Respiratory
Follow up times

P7:

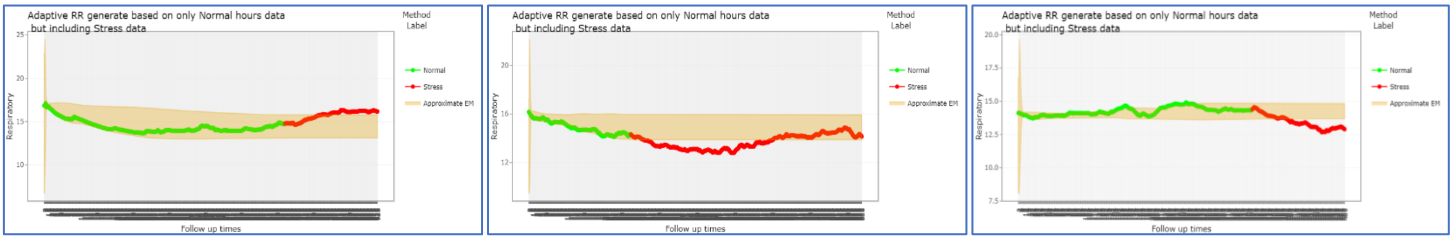

P8:

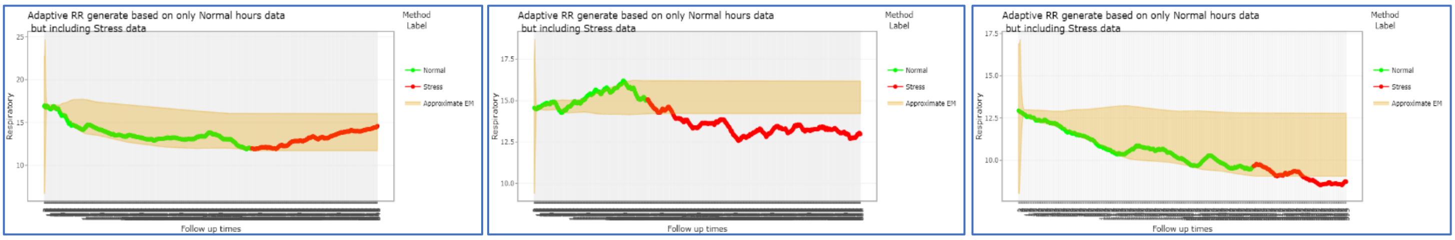

P9:

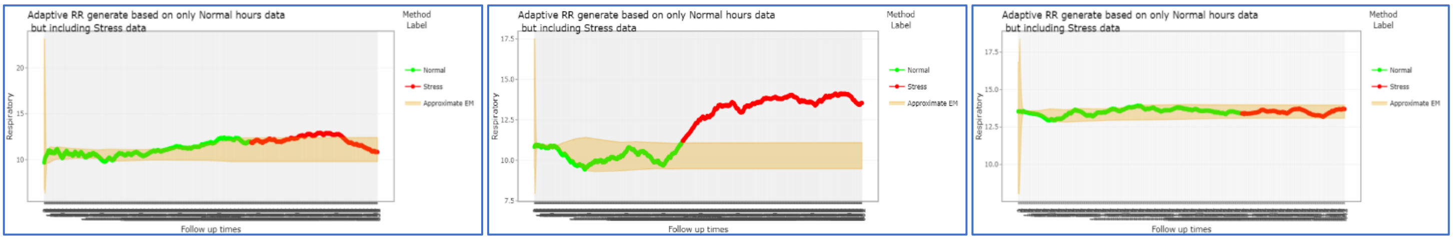

P10:

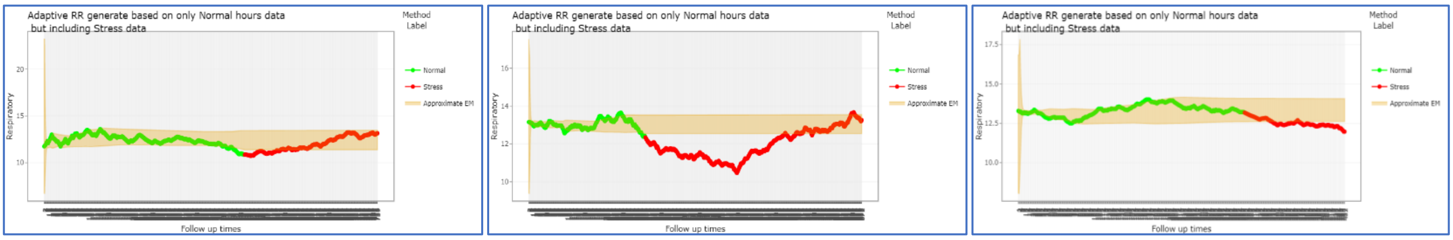

P11:

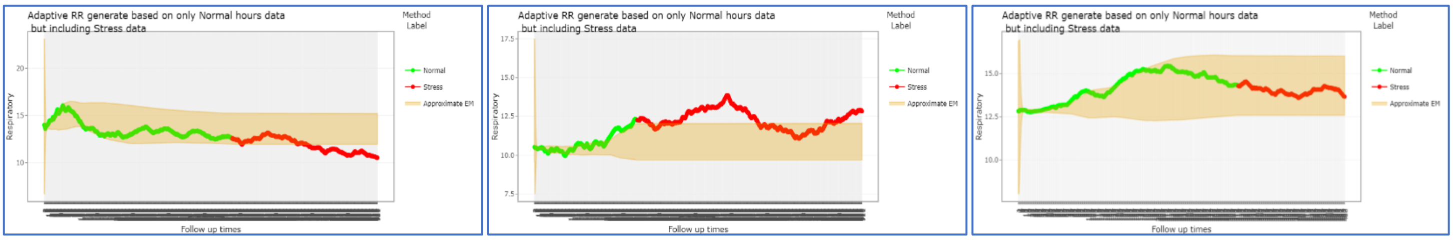

P12:

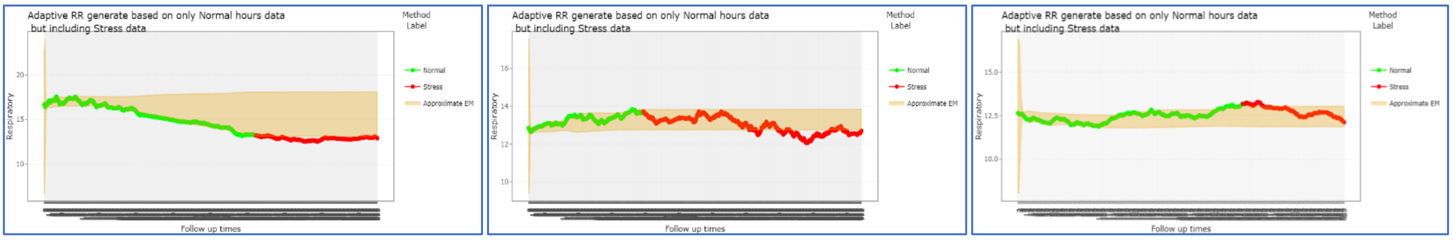

P13:

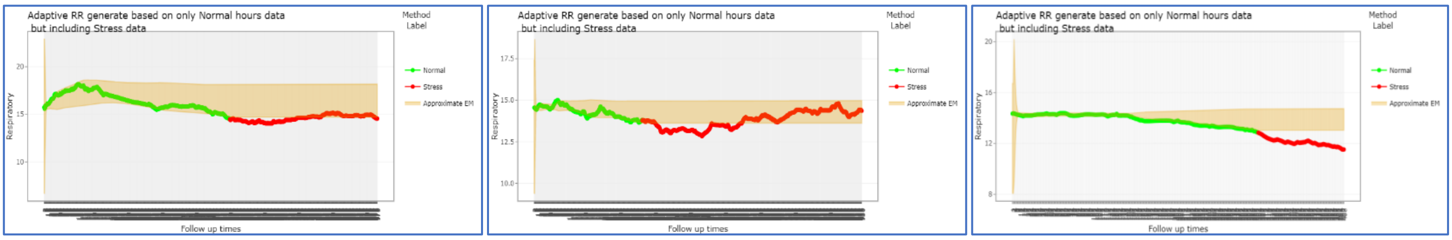

P14:

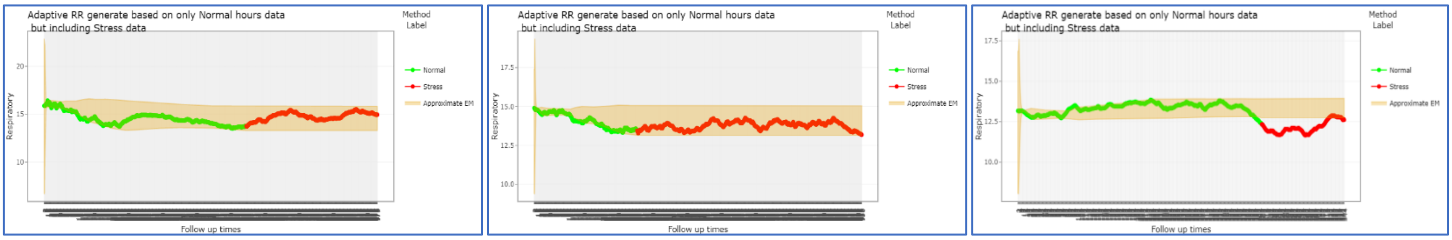

P15:

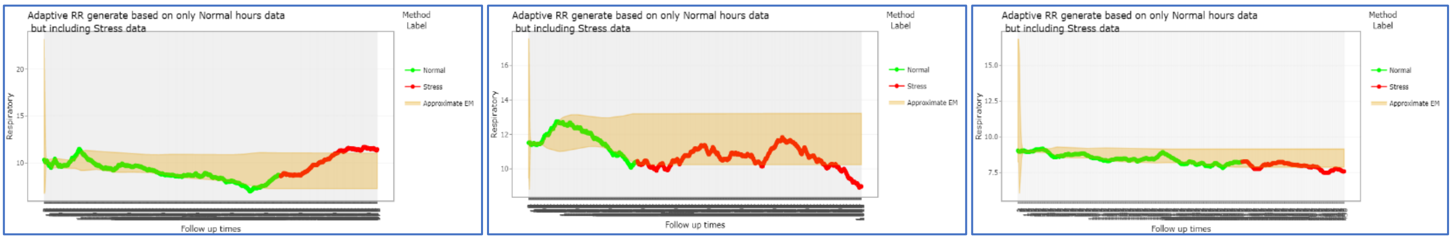

P16:

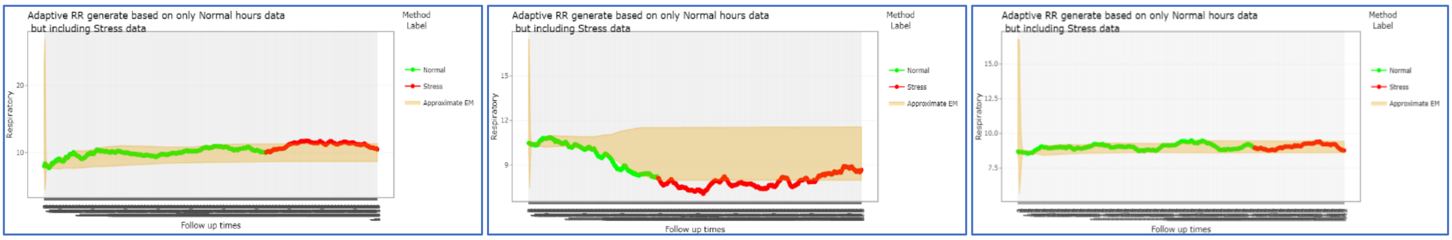

P17:

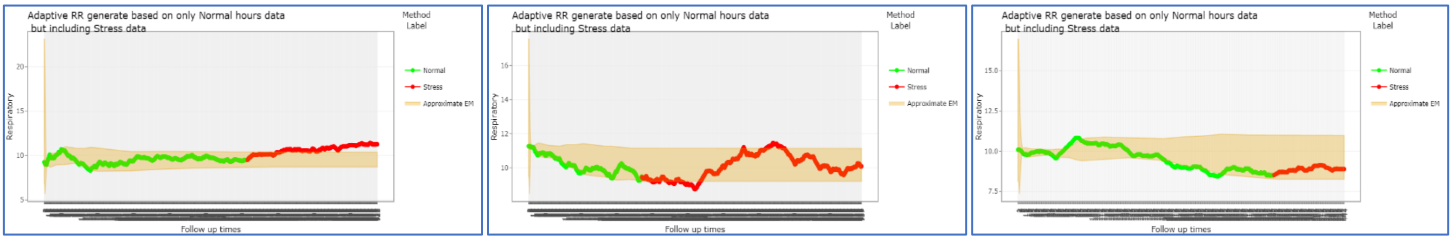

P18:

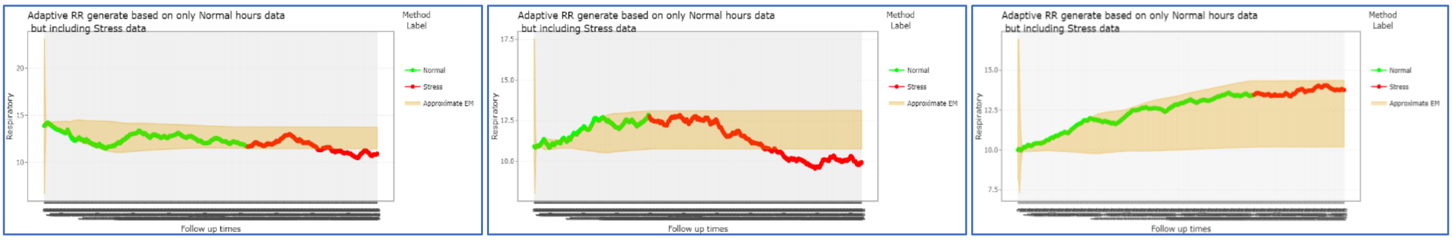

P19:

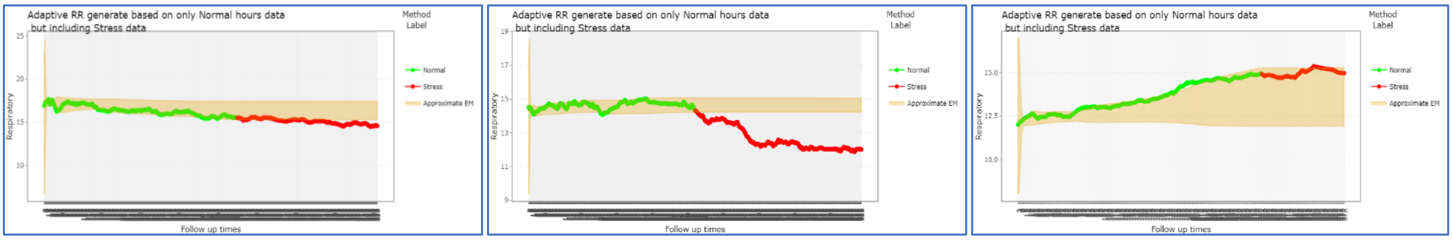

P20:

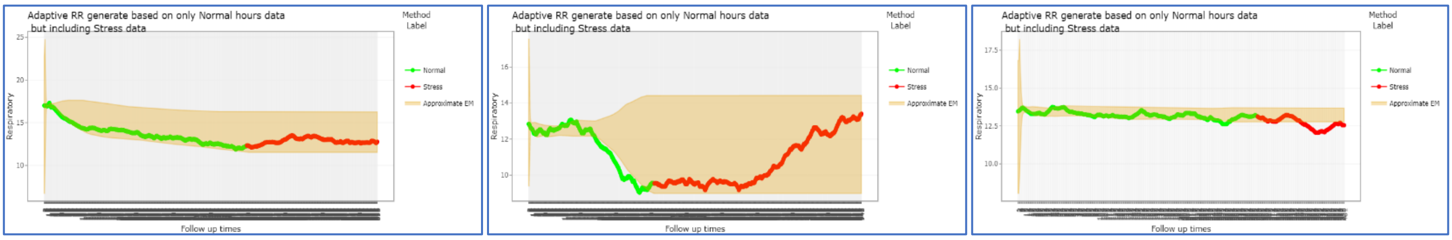

P21:

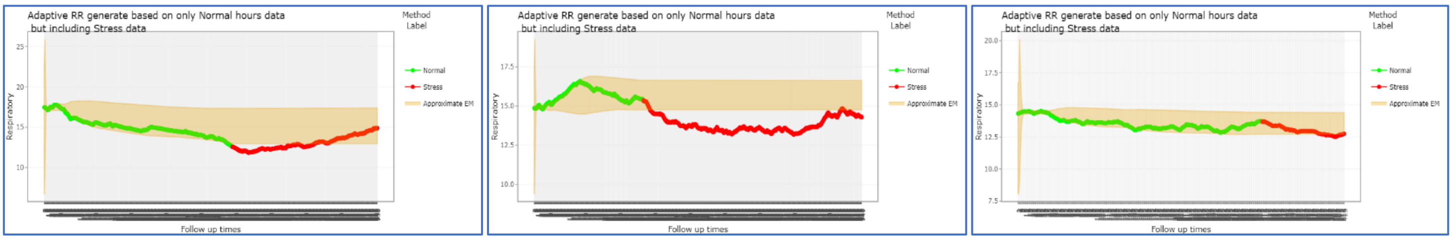

P22:

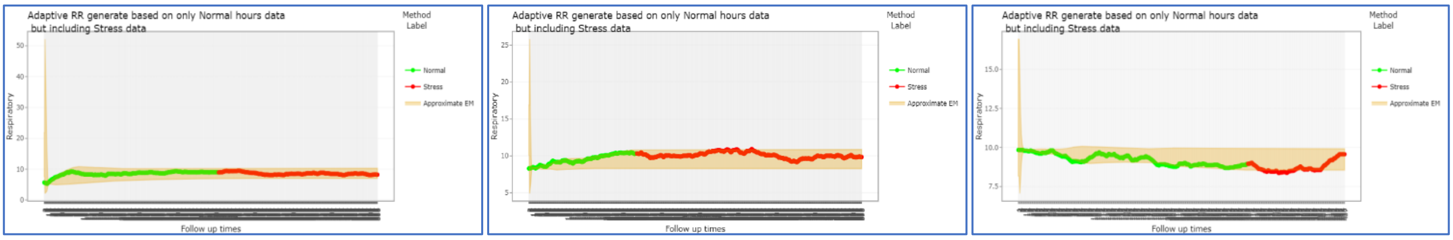

P24:

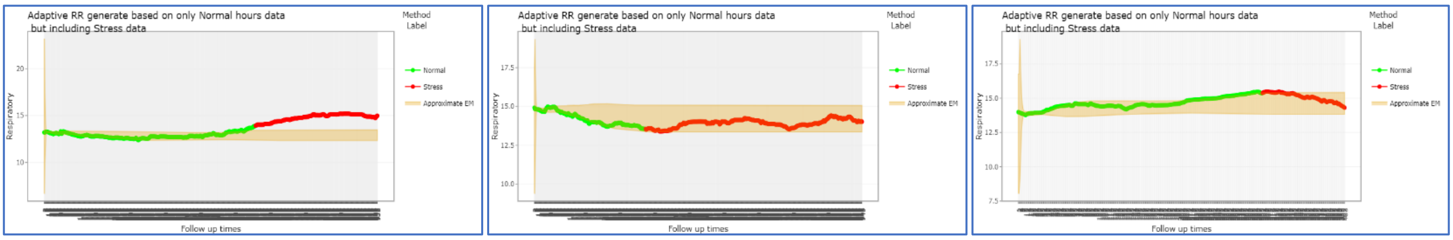

P25:

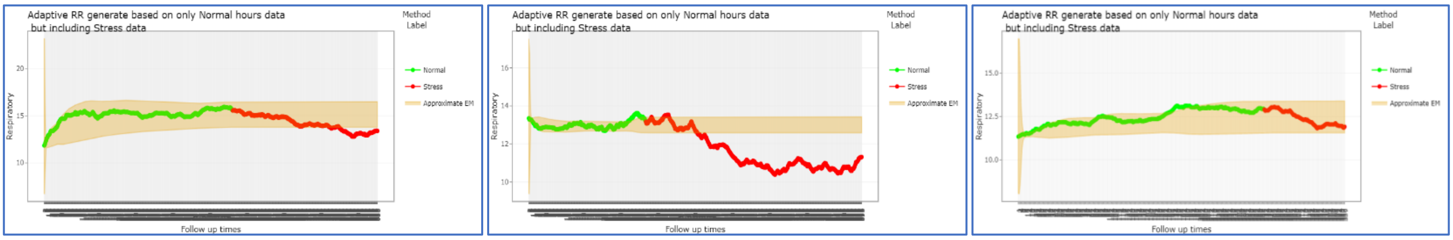

P26:

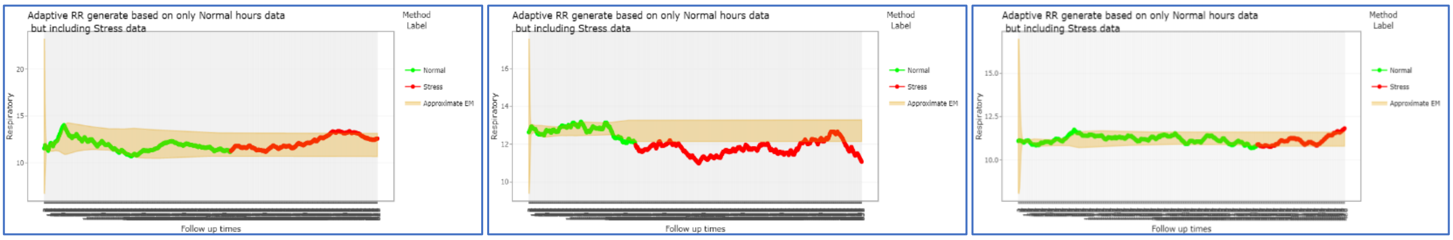

P27:

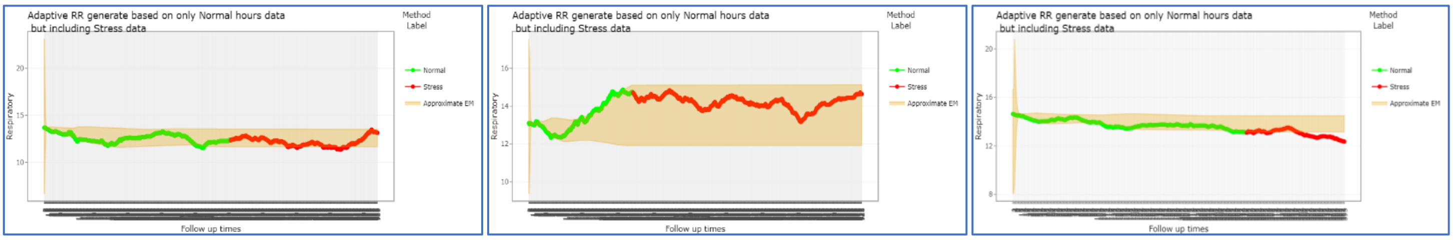

P28:

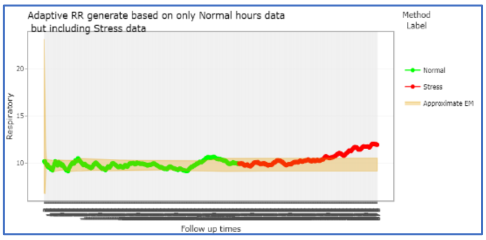

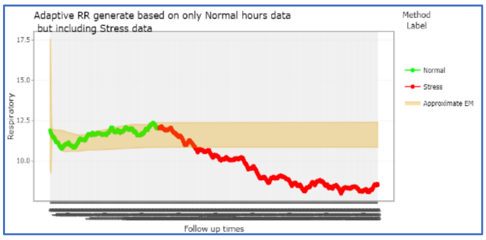

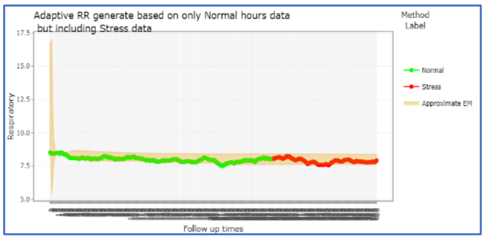

P29:

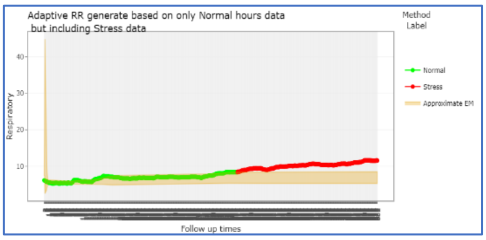

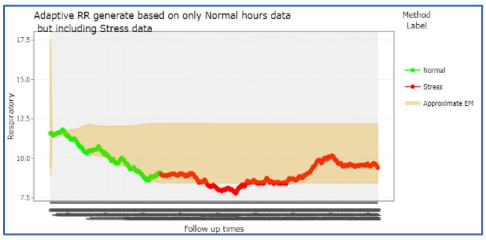

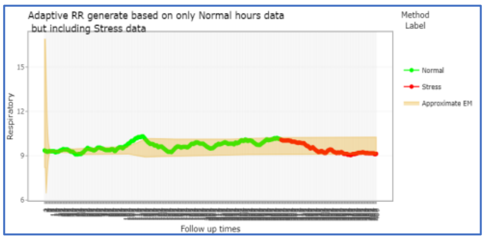

P30:

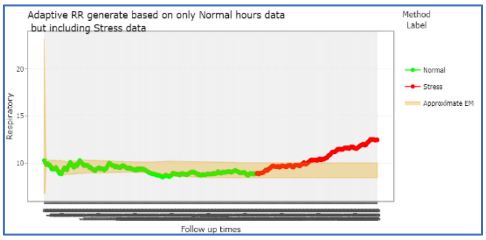

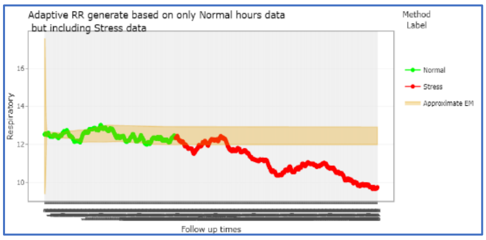

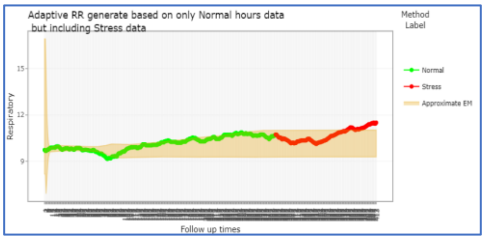

P31:

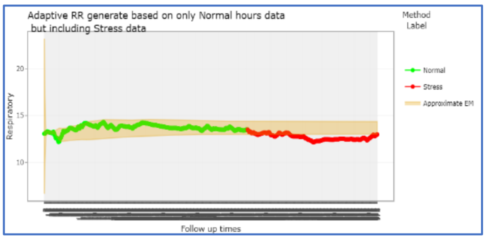

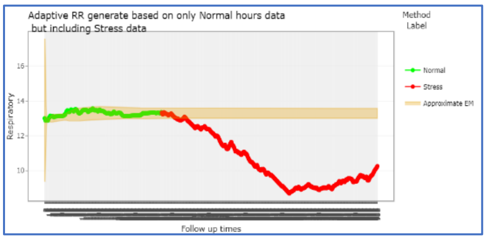

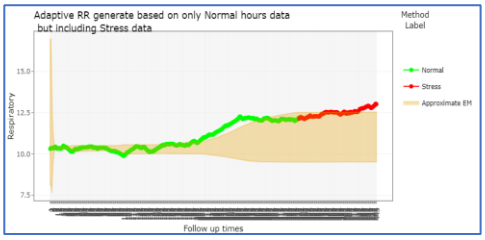

P32:

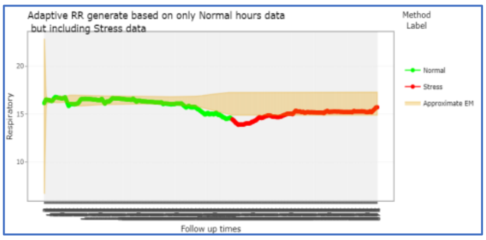

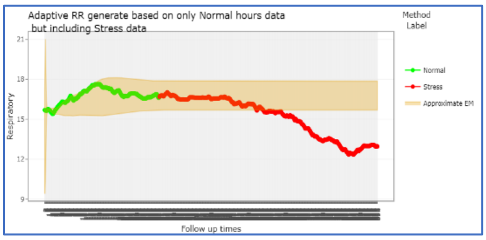

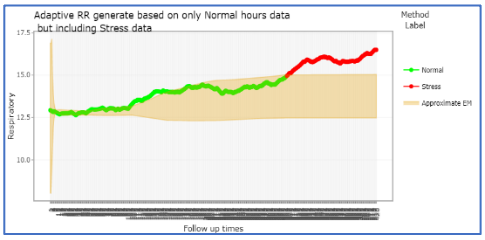

P33:

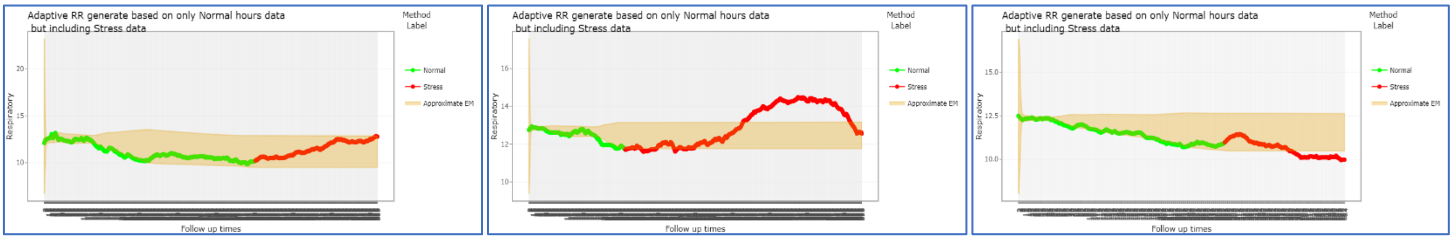

Adaptive RR generate based on only Normal hours data
but including Stress data
Method
Label
Normal
Stress
Approximate EM
Respiratory
20
15
10
Follow up times
Adaptive RR generate based on only Normal hours data
but including Stress data
Method
Label
Normal
Stress
Approximate EM
Respiratory
16
14
12
10
Follow up times
Adaptive RR generate based on only Normal hours data
but including Stress data
Method
Label
Normal
Stress
Approximate EM
Respiratory
15.0
12.5
10.0
Follow up times

P34:

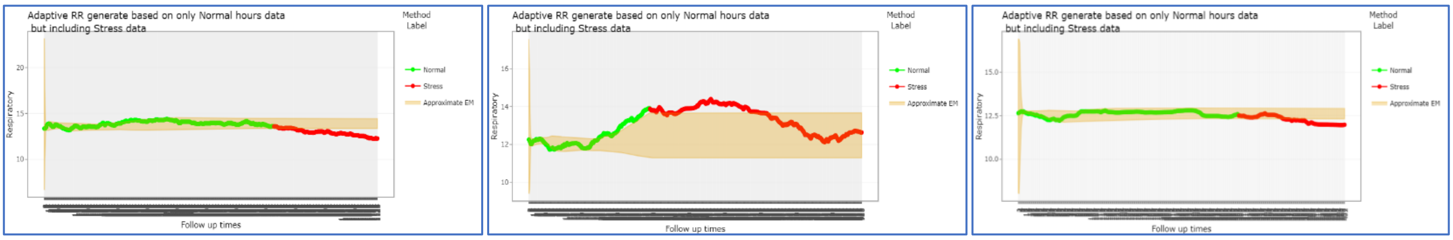

Adaptive RR generate based on only Normal hours data
but including Stress data
Method
Label
Normal
Stress
Approximate EM
Respiratory
20
15
10
Follow up times
Adaptive RR generate based on only Normal hours data
but including Stress data
Method
Label
Normal
Stress
Approximate EM
Respiratory
16
14
12
10
Follow up times
Adaptive RR generate based on only Normal hours data
but including Stress data
Method
Label
Normal
Stress
Approximate EM
Respiratory
15.0
12.5
10.0
Follow up times

P35:

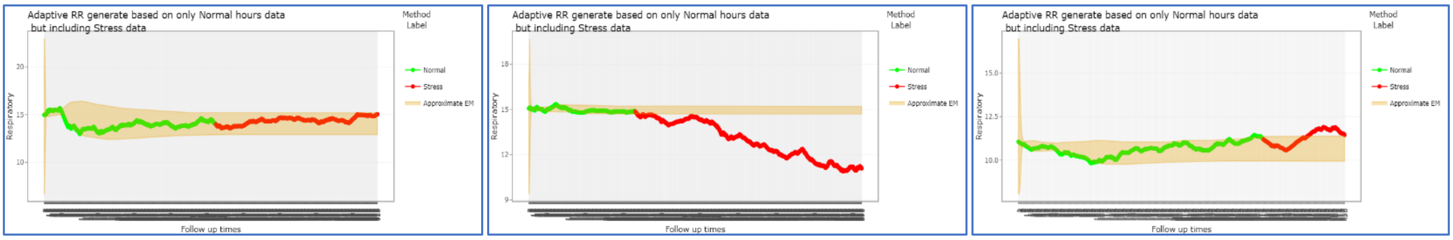

Adaptive RR generate based on only Normal hours data
but including Stress data
Method
Label
Normal
Stress
Approximate EM
Respiratory
20
15
10
Follow up times
Adaptive RR generate based on only Normal hours data
but including Stress data
Method
Label
Normal
Stress
Approximate EM
Respiratory
18
15
12
9
Follow up times
Adaptive RR generate based on only Normal hours data
but including Stress data
Method
Label
Normal
Stress
Approximate EM
Respiratory
15.0
12.5
10.0
Follow up times
