# Supplementary figures and images for: Stress Monitoring Using Wearable Sensors: A Pilot Study and Stress-Predict Dataset
Source: Sensors (Basel). 2022 Oct 24;22(21):8135. doi: 10.3390/s22218135 (PMC9654418; doi:10.3390/s22218135)

P2:

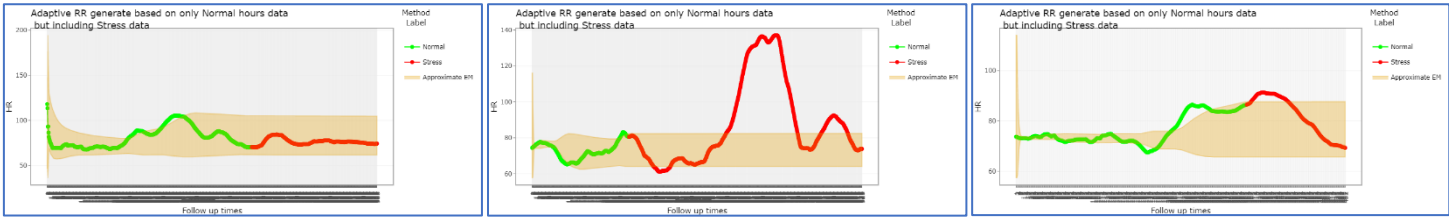

P3:

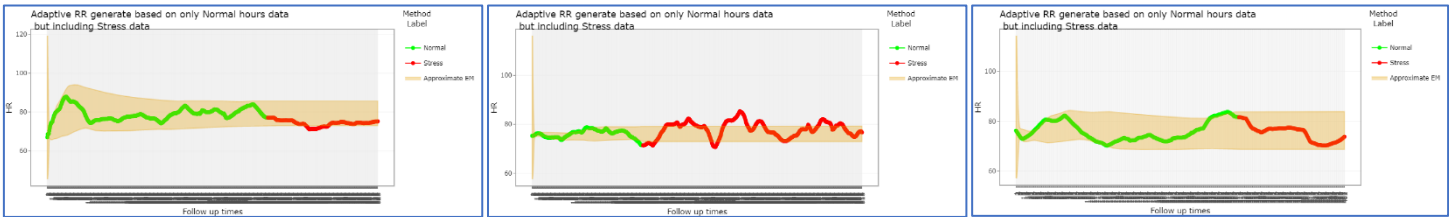

P4:

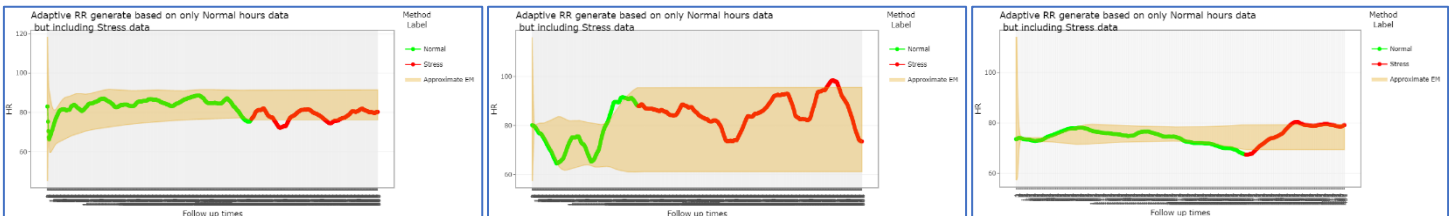

P5:

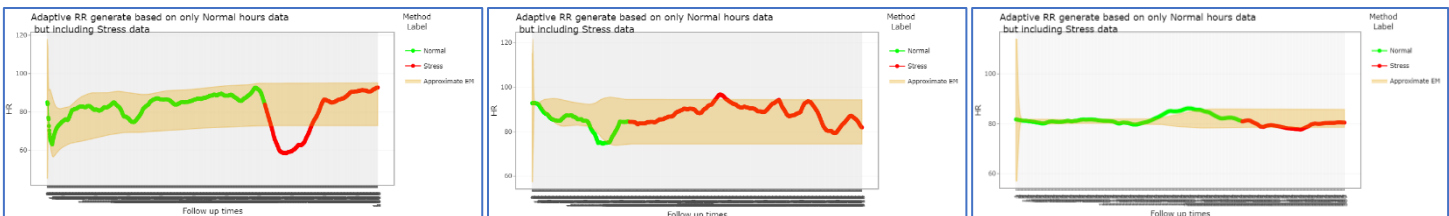

P6:

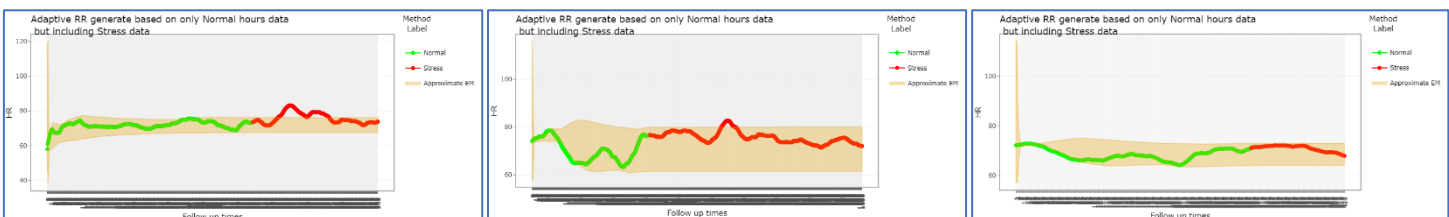

P7:

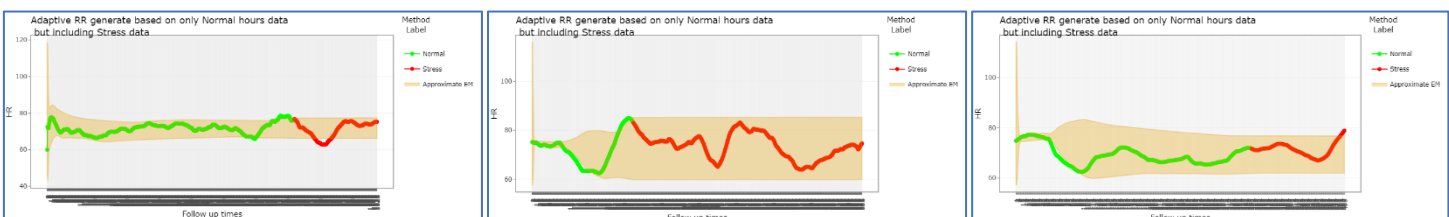

P8:

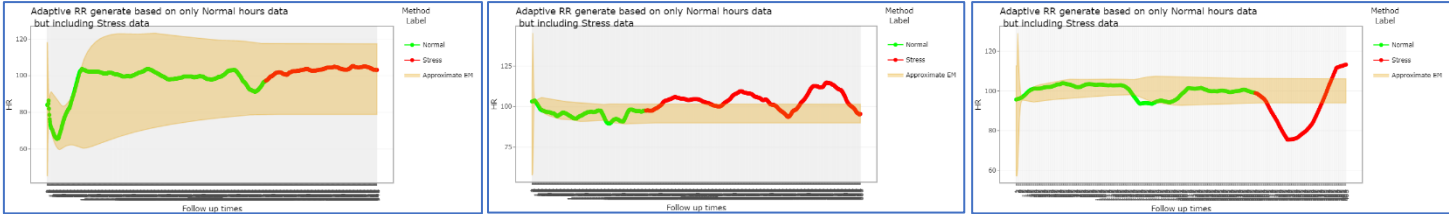

P9:

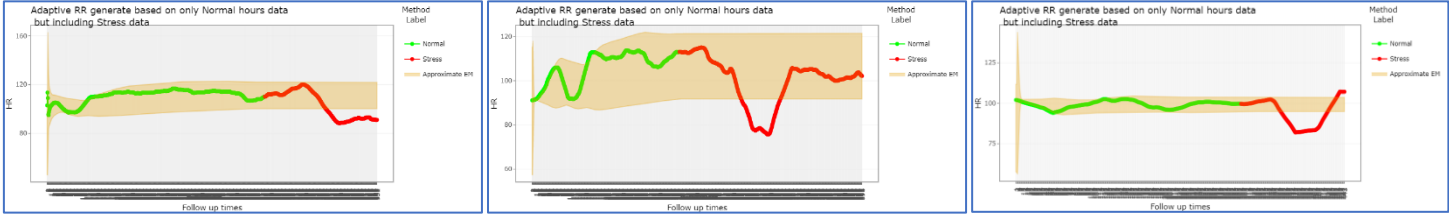

P10:

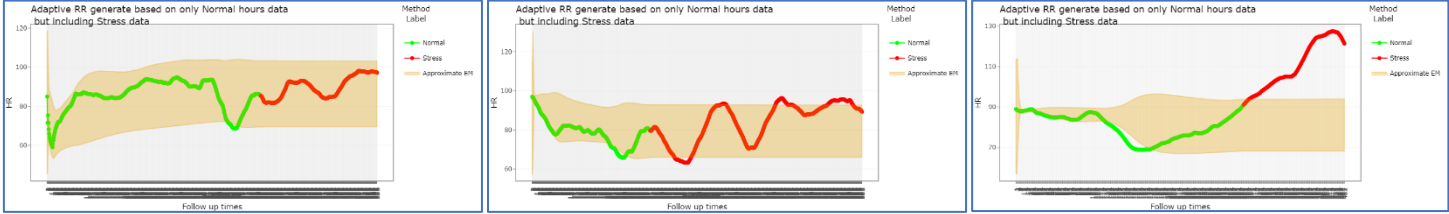

P11:

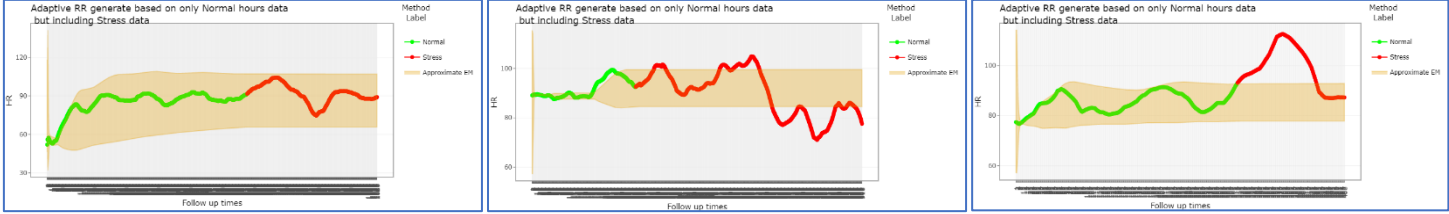

P12:

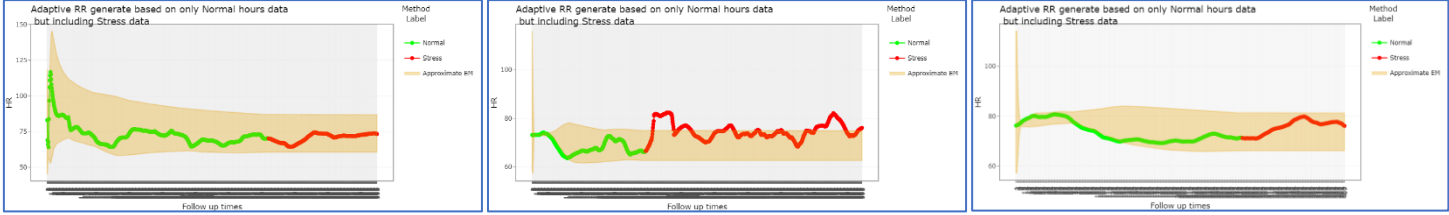

P13:

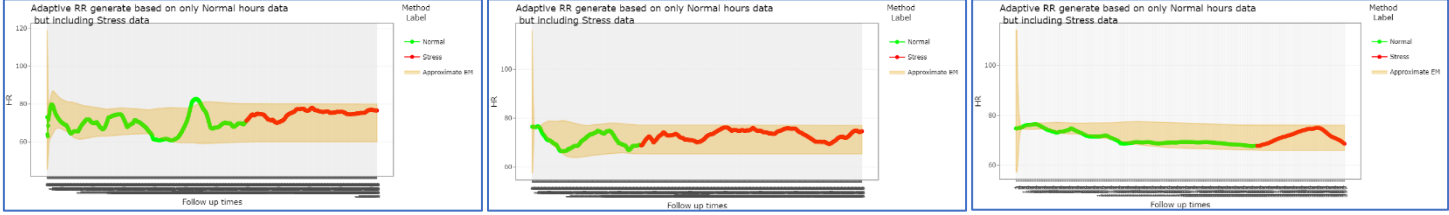

P14:

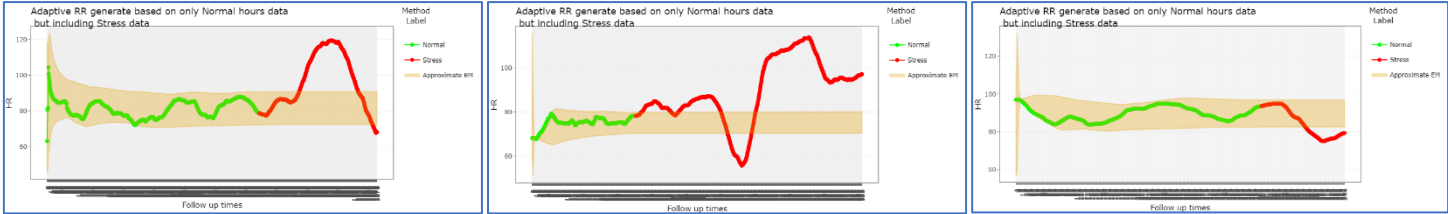

P15:

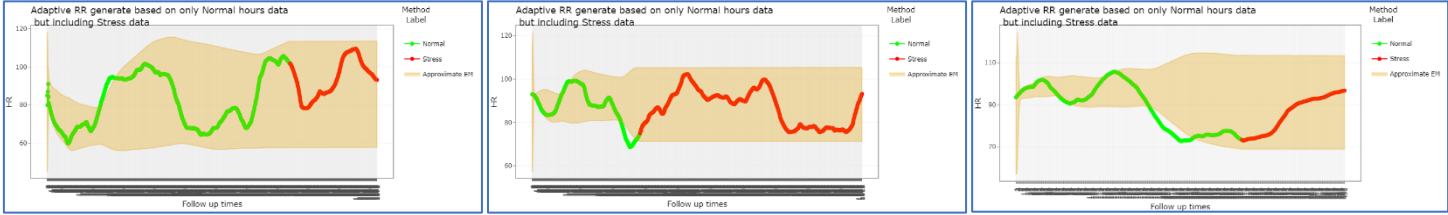

P16:

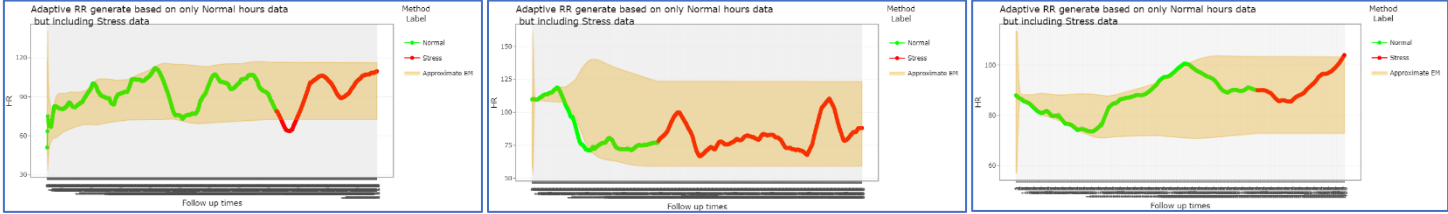

P17:

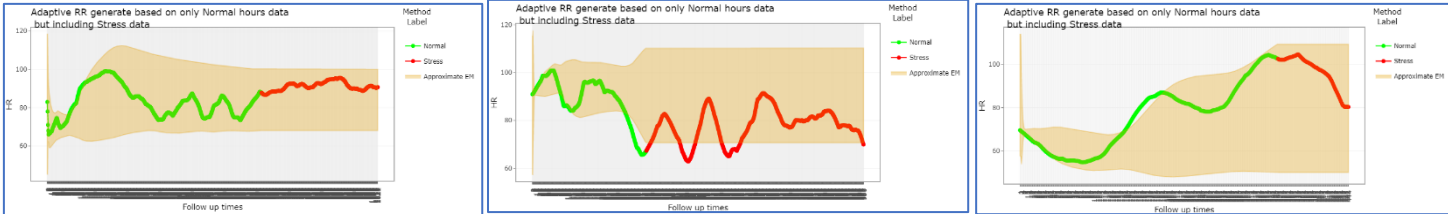

P18:

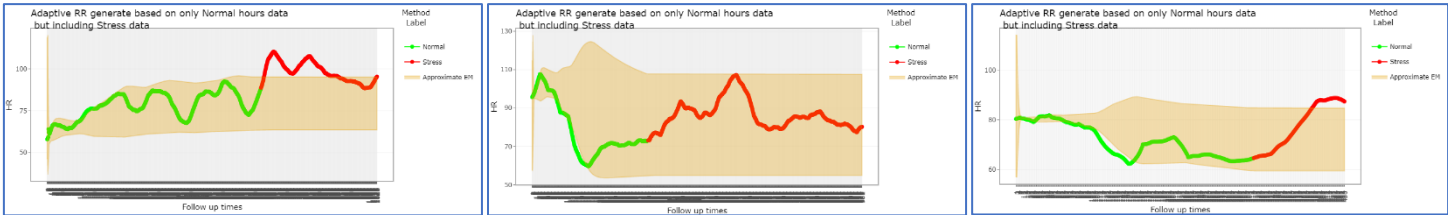

P19:

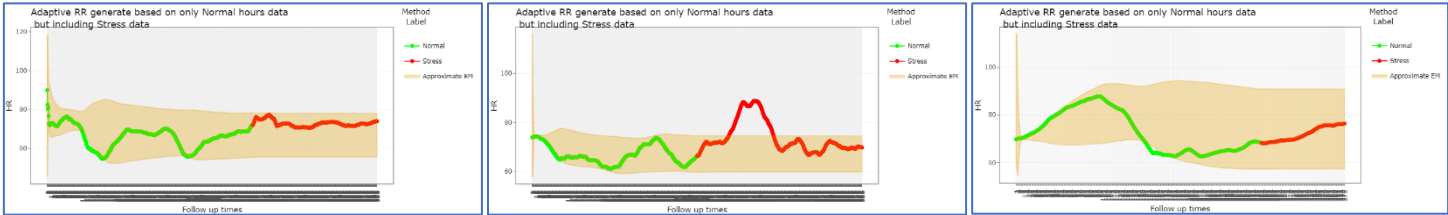

P20:

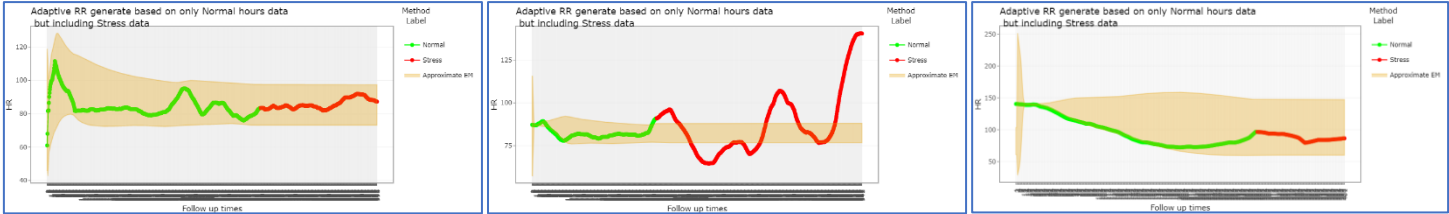

P21:

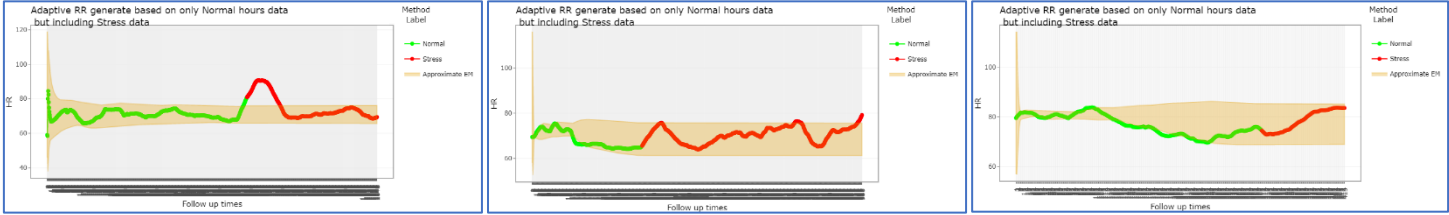

P22:

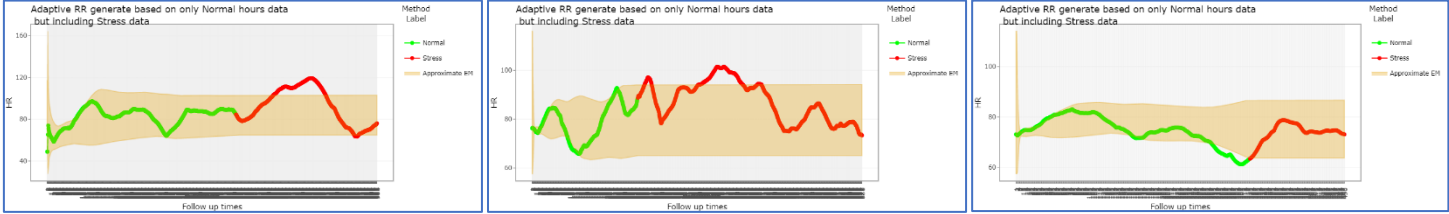

P24:

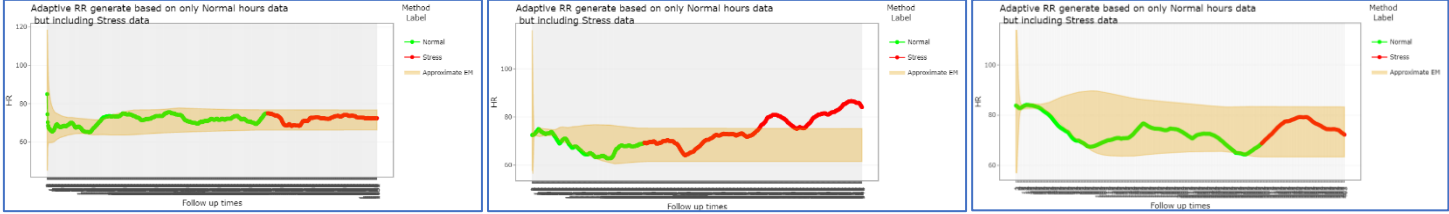

P25:

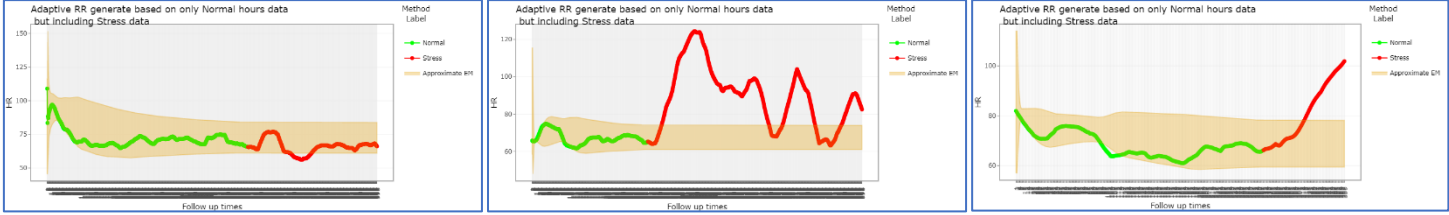

P26:

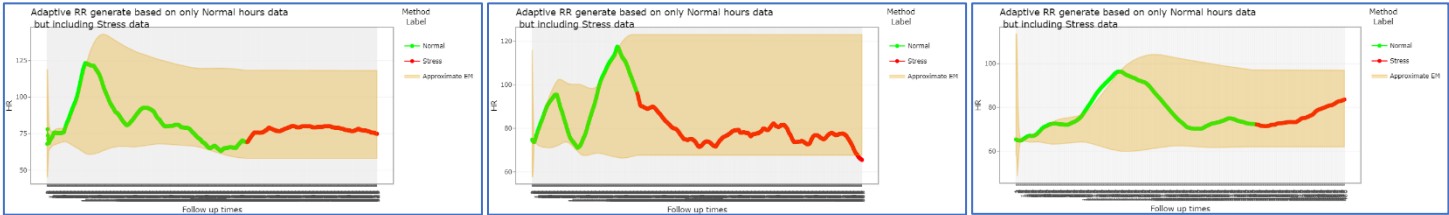

P27:

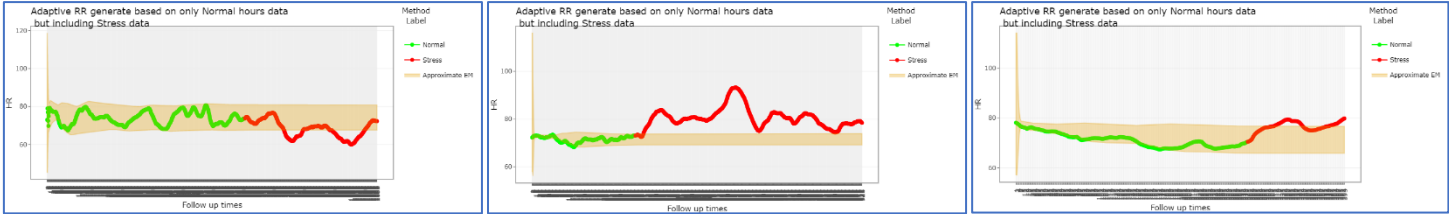

P28:

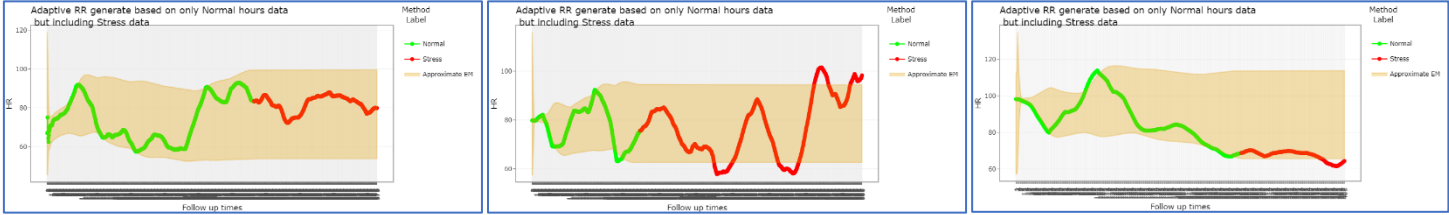

P29:

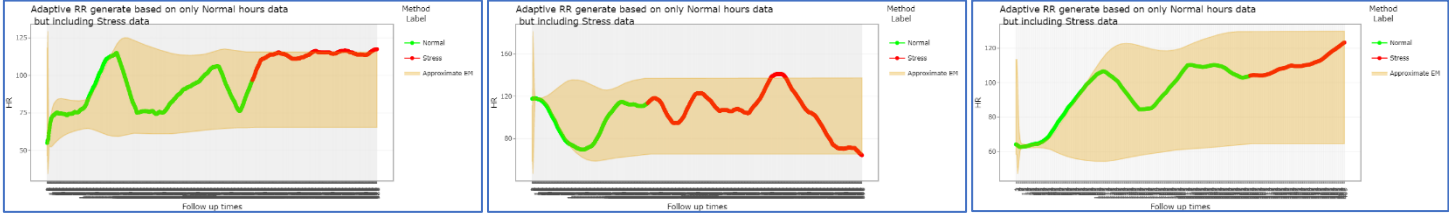

P30:

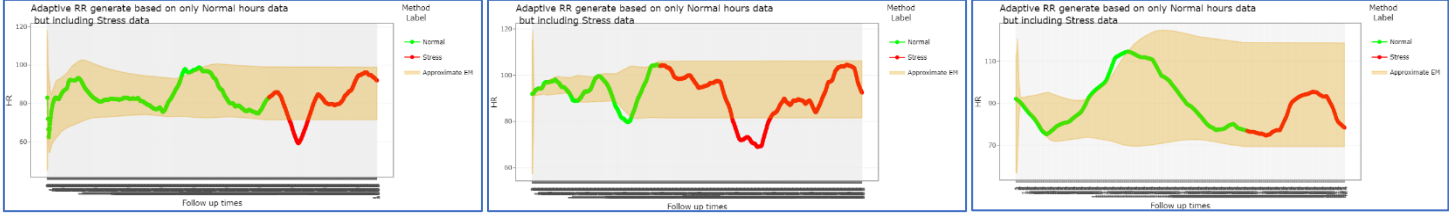

P31:

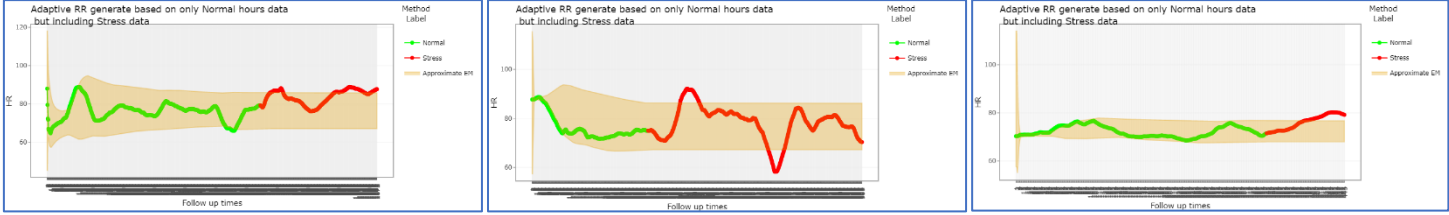

P32:

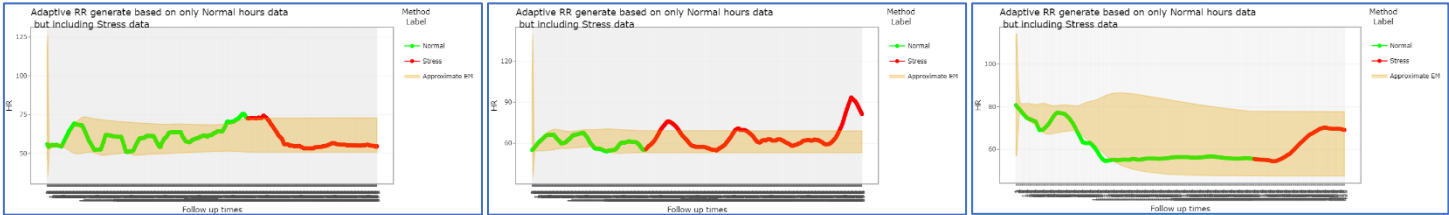

P33:

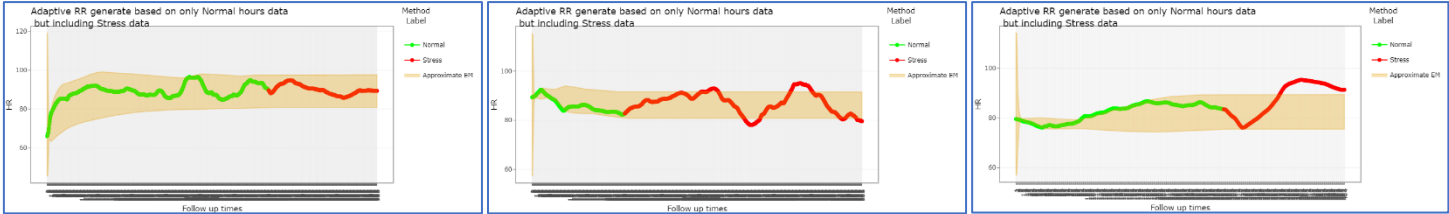

P34:

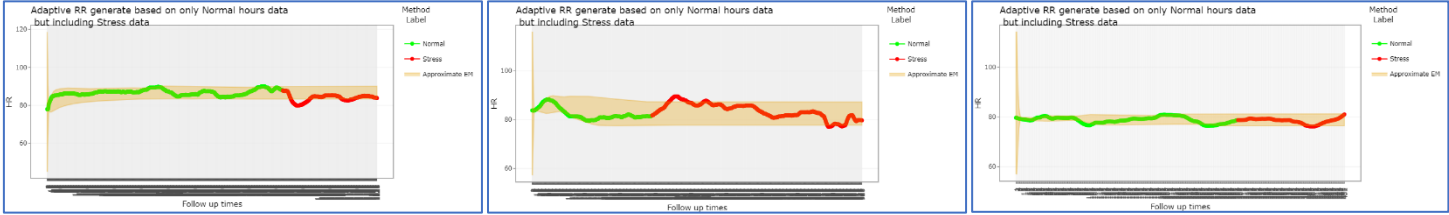

P35:

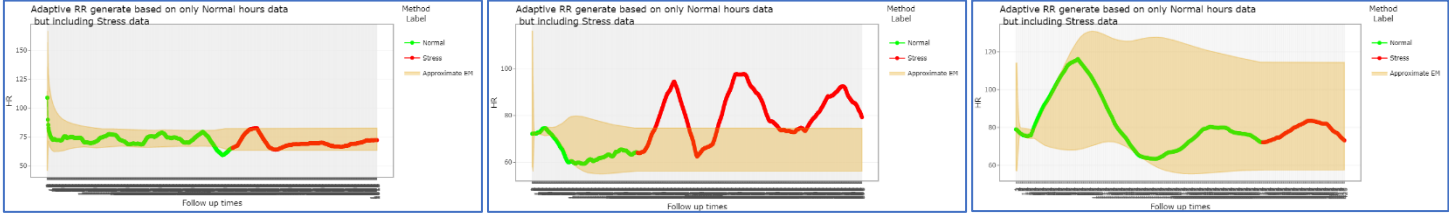

Supplement: Supplementary file 1 [file sensors-22-08135-s001.zip › HR_Plot.pdf]
